# Effects of a weight management program delivered by social media on weight and metabolic syndrome risk factors in overweight and obese adults: A randomised controlled trial

**DOI:** 10.1371/journal.pone.0178326

**Published:** 2017-06-02

**Authors:** Monica Jane, Martin Hagger, Jonathan Foster, Suleen Ho, Robert Kane, Sebely Pal

**Affiliations:** 1 School of Public Health, Curtin University, Perth, Australia; 2 School of Psychology and Speech Pathology, Curtin University, Perth, Australia; 3 School of Applied Psychology and Menzies Health Institute Queensland, Griffith University, Brisbane, Australia; 4 Faculty of Sport and Health Sciences, University of Jyväskylä, Jyväskylä, Finland; 5 Department of Physical Education, Hong Kong Baptist University, Kowloon Tong, Hong Kong; 6 Neurosciences Unit, Health Department of WA, Perth, Australia; Weill Cornell Medical College Qatar, QATAR

## Abstract

**Introduction:**

The aim of this project was to evaluate the effectiveness of using social media to augment the delivery of, and provide support for, a weight management program delivered to overweight and obese individuals during a twenty four week intervention.

**Methods:**

Participants randomly divided into either one of two intervention groups or a control group. The two intervention groups were instructed to follow identical weight-management program. One group received the program within a Facebook group, along with a support network with the group, and the other intervention group received the same program in a booklet. The control group was given standard care. Participants’ weight and other metabolic syndrome risk factors were measured at baseline and at weeks 6, 12, 18 and 24.

**Results:**

The Facebook Group reported a 4.8% reduction in initial weight, significant compared to the CG only (p = 0.01), as well as numerically greater improvements in body mass index, waist circumference, fat mass, lean mass, and energy intake compared to the Pamphlet Group and the Control Group.

**Conclusions:**

These results demonstrate the potential of social media to assist overweight and obese individuals with respect to dietary and physical activity modifications for weight management, and justify further research into the inclusion of social media in clinical weight management programs. It is anticipated that social media will provide an invaluable resource for health professionals, as a low maintenance vehicle for communicating with patients, as well as a source of social support and information sharing for individuals undergoing lifestyle modifications.

## Introduction

Since 1980 world-wide rates of obesity has doubled [1]. According to the World Health Organisation, obesity is now a global epidemic [2] and is responsible for an estimated 2.8 million deaths per year [3]. This is despite the recognition of the importance of this issue among health professionals [4–7] as well as increasing awareness of obesity within the wider community [8]. Previous public health weight management strategies have not had the desired impact and newer approaches need to be considered. Excessive weight gain is strongly related to socio-environmental changes that promote the consumption of high energy diets and reduced physical activity [1, 7]. This is particularly so for the socioeconomically disadvantaged [9]. Obesity increases the risk of cardiovascular disease, stroke, type 2 diabetes and some cancers [1, 5, 7]. On the other hand, supportive environments and communities can influence dietary and lifestyle choices by making healthy choices available, affordable and accessible [1]; these approaches could therefore be used to treat and prevent obesity.

Weight loss can reduce the cardio-metabolic risk factors associated with obesity [10, 11]. However, many dieters have difficulty with ongoing weight loss maintenance [12]. In an effort to overcome this problem researchers have found that implementing multifactorial weight management programs are more likely to achieve clinically meaningful weight loss results [7], as opposed to following weight loss instructions only. Supplementary strategies include frequent appointments with health professionals, cognitive behavioural therapy, use of supplements and group support sessions [7, 9, 13]. Similarly, individuals have better health outcomes if they are well supported socially [14, 15]; this includes better weight loss outcomes [16, 17]. However, many individuals do not have adequate support while attempting weight loss for a number of reasons [15].

The social aspect may be an important factor that contributes to the effectiveness of group weight management programs. Some studies have found that group weight management programs result in better weight loss outcomes when compared to individual treatment [18, 19]. Group programs are also a more cost-effective option to individual programs [18, 20].

Recent developments in internet and communication technologies may offer health promoters a novel platform for group weight management programs. Internet-based health intervention trials focusing on behaviour change have incorporated a social element using chat rooms or discussion boards, with many of these interventions providing feedback via health professionals or mobile monitoring devices [21, 22]. Internet-mediated social networking sites improve upon these features; already studies have shown that networked members can provide each other with support [23, 24]. This technology also offers new avenues for information sharing [25], so that information and member support are accessible at home or away 24-hours a day seven days a week, at the convenience of members.

Economic analysis shows that internet-delivered weight management programs costs less per person–*and* per kilogram lost—than an in-person program [26]. Social media may be an even less expensive avenue, particularly if an existing platform is used (e.g. Facebook). This approach has the added convenience of direct access to existing online social networks [27, 28]. In addition, the cost-effectiveness and large scale online connectivity of social media has the potential to assist individuals on low incomes or in geographically remote communities [29] to access support while following a weight management program. Furthermore the increased interactivity of social media (used in conjunction with personal profiles) may provide a friendlier setting that enhances online intervention outcomes.

Few studies have examined the value of using a social media platform like Facebook for weight management, and no studies have been undertaken to date that promote dietary *and* physical activity modifications with the only feedback being that which can be derived from other study participants, or targeting a particular condition (eg diabetes), age group or gender [21, 22].

The aim of the current study was to measure changes to weight and other obesity-related disease risk factors in overweight and obese participants when a weight management program was delivered using social media, compared to the same program presented in written information only, over a period of twenty four weeks. It was hypothesised that compared to the Control Group and Pamphlet Group, the Facebook Group would experience greater improvements in weight and other metabolic syndrome risk factors over the 24 week intervention period. In particular, the changes to weight in the were hypothesised to be 2% of initial body in the Pamphlet Group, and 9% of initial body weight in the Facebook Group, compared to the Control Group.

## Methods

### Participants

Overweight and obese individuals with a body mass index (BMI) between 25–40 kg/m^2^ and aged between 21 and 65 years were recruited from the Perth community via advertisements in the West Australian Newspaper and Community Newspapers between 2 July and 11 November 2014. Participants were required to have access to a computer, laptop, tablet or Smartphone. Two hundred and eighty four respondents were screened by telephone interview, and one hundred and thirty seven individuals were found to be eligible. Exclusion criteria included smoking, lipid lowering medication, use of steroids and other agents that may influence lipid metabolism, use of warfarin, diabetes mellitus, hypo- and hyperthyroidism, cardiovascular events within the last 6 months, major systemic diseases, gastrointestinal problems, proteinuria, liver disease, renal failure, weight fluctuations over the past 6 months, vegetarianism and participation in any other clinical trials within the last 6 months. These measures were in place to ensure harm minimisation and to prevent the introduction of potential confounders. This study was conducted according to the ethical guidelines provided by the National Health and Medical Research Council. The original study protocol was approved by the Curtin University Human Research Ethics Committee (approval no. HR90/2014) prior to trial commencement, as reported elsewhere [30]. In addition, the amendments to the original study protocol explained in this work also received approval from the Curtin University Human Research Ethics Committee prior to trial commencement. All identifiable information collected from participants was coded. All participants provided signed, written informed consent. This trial was registered with the Australian New Zealand Clinical Trials Register (trial registration no.: ACTRN12614000536662).

### Study design

The original protocol consisted of a 12-week intervention period with a 12-week follow-up, as previously reported [30]. The current study was an adaptation of the original intervention and was conducted as a 24-week, three-armed, randomised, controlled, parallel design (without follow-up) investigation [30]. Recruited participants were enrolled and assigned a three-digit number in chronological order by the study co-ordinator. Participants were then randomised to one of the three groups by block randomisation according to age and gender, using online research randomising software [31] (i.e. random number generator). Participants were blinded; randomisation and group allocation was undertaken by the study co-ordinator.

### Interventions

Prior to trial commencement participants attended information sessions at Curtin University where full details of the study were explained, which included a brief overview of the treatment lasting approximately half an hour, according to group allocation. The Control Group (CG) were instructed to follow the Australian Government dietary guidelines [32] as well as the *National Physical Activity Guidelines for Adults* [33] as standard care. Both the Pamphlet Group (PG) and the Facebook Group (FG) were instructed to follow the *Total Wellbeing Diet* developed by the Commonwealth Scientific and Industrial Research Organisation, following rigorous scientific testing and proven to result in weight loss [34]. This program is an energy-reduced, low fat, lower carbohydrate, higher protein diet, as explained in greater detail elsewhere [30]. Both the PG and the FG received a condensed version of the diet, which included detailed information and instructions, compiled from excerpts from both the *Total Wellbeing Diet Book 2* [35] and *Total Wellbeing Diet Recipes on a Budget* [36] (with permission from Penguin Publishing). The PG received the information in written form as a booklet, while the FG received identical information contained within the booklet but with pages as snapshots posted within the ‘secret’ (i.e. closed and hidden from the general Facebook population) Facebook group. (See; S2 File. Trial Protocol Part 1. Intervention Program.) In addition to information from the *Total Wellbeing Diet*, participants in both intervention groups were also issued with a pedometer (G Sensor 2025 Accelerometer, Walk with Attitude Australia) and instructed to achieve a target of 10,000 steps per day (as recommended in the *Total Wellbeing Diet* program). The FG were given additional information on how to use the Facebook group to access the weight management program, encouraged to interact with one another, and had the rules of polite interaction with other group members were explained to them. Following the completion of the information sessions, FG participants were invited to join the Facebook group by the study co-ordinator, who acted as the administrator of the group. Participants in all groups were given the necessary materials at the conclusion of their baseline clinic appointments and instructed to commence the intervention forthwith. None of the participants were given any further external weight management guidance during the trial by the study coordinator. The only access the FG had to the program was the information posted on Facebook. In addition, the study co-ordinator posted to the Facebook group once per week to the Facebook group over the 24 week intervention. (More detailed information can be found the additional file with the title of S3 File. Trial Protocol Part 2. Project Outline.)

### Assessments

The primary outcome for this trial was weight. The secondary outcome measures were blood pressure waist and hip circumference, fasting blood glucose, lipids and insulin, dietary intake, physical activity and step count (the latter for the PG and the FG only). Participants attended clinical appointments at Curtin University in the fasted state at baseline, and at weeks 6, 12, 18 and 24, (with no follow-up appointment. This appointment schedule is an update of the original study protocol consisting of appointments at baseline, weeks 6 and 12, with a follow-up appointment 12 weeks after the end of the initial 12 week intervention [30]. See S1 Fig for the up-to-date schedule of outcome measures). At these appointments, weight was measured in light clothing without shoes (UM-018 Digital Scales; Tanita Corporation, Tokyo, Japan). Differences in weight at each time point were calculated per individual as a percentage of total baseline body weight. Height (baseline only) was measured using a stadiometer (26SM 200 cm SECA, Hamburg, Germany) without shoes. Waist circumference was measured in the standing position at the narrowest area between the lateral lower rib and the iliac crest, and hip circumference was measured at the widest area across the buttocks. Fasting blood glucose measurements were taken using the Accu-Chek® Performa glucometer and lancing device (Roche Diagnostics). (Arterial stiffness was removed from the list of outcome measures reported in the original study protocol [30]. See S1 Fig for the up-to-date schedule of outcome measures).

At baseline, weeks 12 and 24 blood pressure was measured with an automated, calibrated sphygmomanometer (Dinamap, Compact T, Critikon, Germany). Lean mass and fat mass was measured in light clothing and without shoes by bioelectrical impedance (using the digital scales already cited), and recorded as a percentage of total body weight per individual. In addition, participants attended their local PathWest Collection Centre to have fasting blood samples taken to measure blood lipids (ie. total cholesterol, triacylglycerols, low density lipoproteins and high density lipoproteins) and blood insulin at baseline, and at weeks 12 and 24. Blood sample analysis was conducted at PathWest Laboratory Medicine, QEII Medical Centre, Nedlands, Western Australia.

Participants were required to return their completed Three-Day Food Records as well as Three-Day Physical Activity Records [37] with three-day step count (PG and FG participants only) at each time point. Energy and macronutrient intakes from the participants’ food records were calculated using Food Works Version 7 (Xyris Software, 2012). Macronutrient intakes were recorded as a percentage of total energy intakes per individual, with the exception of fibre (which was calculated in total grams). Energy expenditure from participants’ physical activity records was calculated using an equation devised for the purpose [37]. (Psychological and behavioural outcome measures were also collected, as indicated in the original study protocol [30], and will be assessed and reported in a future publications in due course. See S4 File. Trial Protocol Part 3. Questionnaires.)

### Statistical analysis

For a three group study with repeated measures and the ability to detect a weight loss difference of 7% of initial body weight (Cohen’s d = 0.4) [38] between the FG and the PG, and an alpha of 0.05 (two-sided), a sample size of 96 achieves 80% statistical power. To allow for an attrition rate of 20%, it was planned to recruit a minimum of 120 participants. Baseline weight (kg) data were assessed for normality, both by study sample and by group, and were found to be slightly positively skewed. Changes in outcome measures relative to baseline were analysed for between group differences at each time point. Generalised Linear Mixed Models was the method of statistical analysis used as it represents a particular class of regression model that is ‘generalised’ in that it can accommodate violations of normality, and ‘mixed’ as it includes both random and fixed effects [39]. In addition, Generalised Linear Mixed Models is less sensitive to participant attrition because it does not rely on participants providing data at every assessment point but uses all the data present at each assessment point, thus reducing sampling bias and the need to replace missing data [39]. (The above explanation forms the rationale for using this method of analysis in favour of the General Linear Methods eg. repeated measures analysis of variance, outlined in the original study protocol [30]). The covariate structure used in the linear mixed models was variance components [39]. This analysis was implemented through SPSS 22.0 (IBM® SPSS® Statistics, New York, NY). Post hoc power analysis was conducted using G*Power [40]. All data were expressed as mean (±SEM), and statistical tests are evaluated at a p-value of .05.

## Results

### Participants

**Fig 1:**
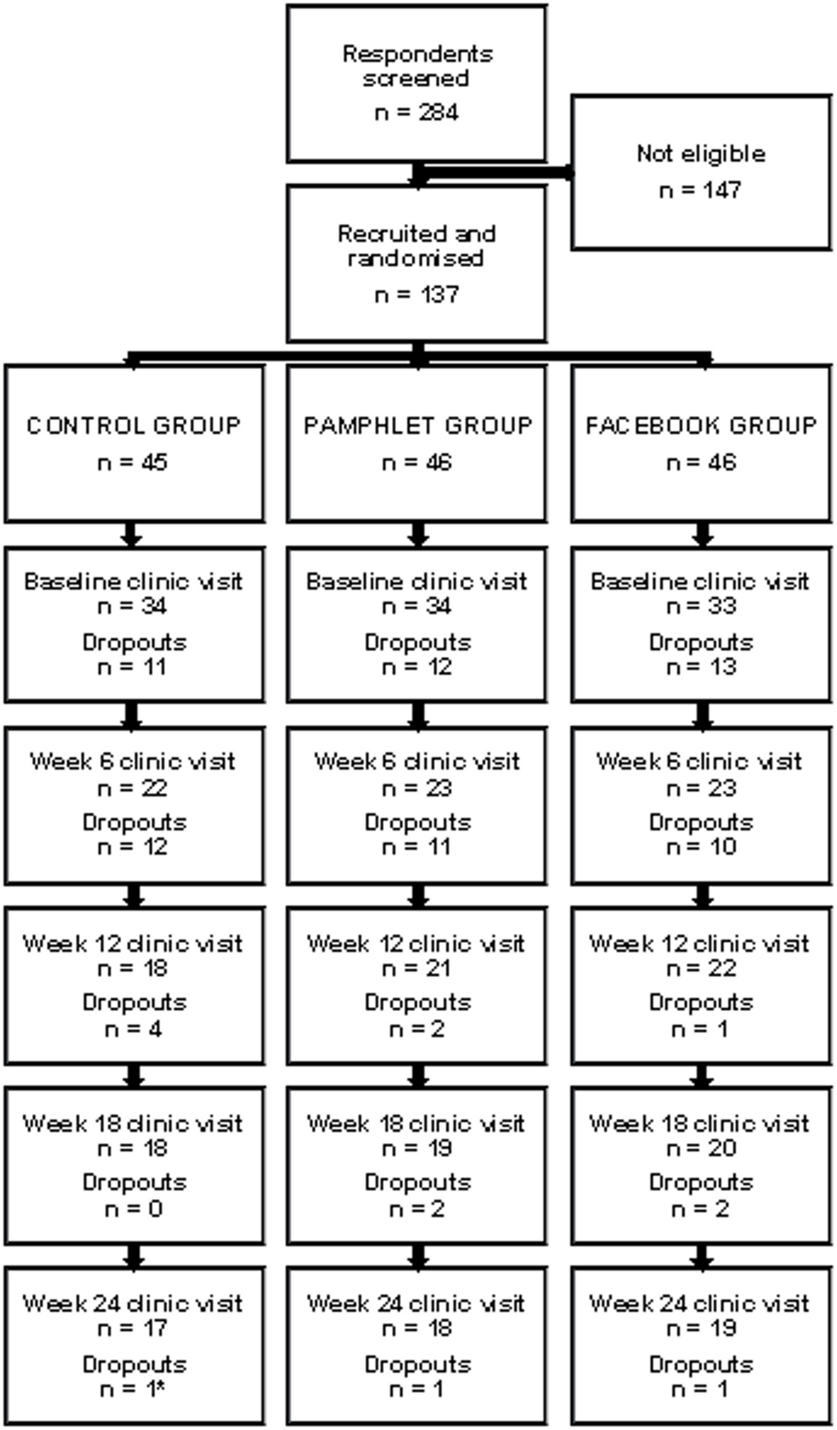
Flow of participants. Reasons for dropouts (n = 82): Did not respond (n = 44); Time constraints (n = 26); Did not like assigned program (n = 5); Unrelated illness (n = 4); Change in personal circumstances (n = 3). No adverse events were recorded. *Data eliminated from the final analysis due to non-compliance.

During the recruitment period, 284 respondents were screened and 137 were found to meet the eligibility criteria (slightly in excess of the required number indicated in the original study protocol [30]; these individuals were invited participate and gave verbal consent to do so. Recruited participants were randomly allocated to one of the three groups as follows CG: *n* = 45; PG: *n* = 46; FG: *n* = 46. One hundred and one participants attended the baseline appointment (CG: *n* = 34; PG: *n* = 34; FG: *n* = 33) at which time they provided written informed consent; among these individuals, 68 participants provided data post-baseline (CG: *n* = 22; PG: *n* = 23; FG: *n* = 23). Fifty six participants completed the full intervention; however, one participant from the CG was eliminated from the final analysis due to non-compliance (CG: *n* = 17; PG: *n* = 18; FG: *n* = 19) (Fig 1).

Data from 67 participants were therefore used for the statistical analysis. Baseline characteristics of all participants that contributed data the analysis are shown (Table 1).

**Table 1:** Baseline characteristics of all participants included in the analysis.

	Control (*n* = 21)*		Pamphlet (*n* = 23)*		Facebook (*n* = 23)*	
	Mean	SEM	Mean	SEM	Mean	SEM
**Gender (m/f)**	4 / 17		2 / 21		4 / 19	
**Age (y)**	50.2	2.4	54.1	2.3	47.0	2.3
**Height (cm)**	165.1	1.5	162.2	1.8	165.3	1.9
**Weight (kg)**	91.5	4.5	86.7	4.2	89.0	3.2
**BMI (kg/m^2^)**	33.3	1.3	32.9	1.3	32.5	1.0
**Waist (cm)**	98.0	2.8	96.1	2.5	96.3	2.4
**Hip (cm)**	115.2	2.9	113.8	2.8	113.0	2.1
**FBG (mmol/L)**	5.8	0.2	6.2	0.3	5.5	0.1
**SBP (mmHg)**	124.3	3.8	126.5	3.5	128.4	4.0
**DBP (mmHg)**	69.3	2.2	69.0	1.4	68.6	1.8
**Insulin (mU/L)**	8.1	0.8	8.8	1.0	9.6	1.2 [20]
**Fat Mass (%)°**	45.5	1.5	45.1	1.5	44.0	1.6
**Lean Mass (%)°**	23.6	0.7	23.7	0.7	24.6	0.8
**TC (mmol/L)**	5.7	0.2	5.8	0.2	5.8	0.2 [20]
**TAG (mmol/L)**	1.2	0.1	1.1	0.1	1.3	0.1 [20]
**LDL (mmol/L)**	3.7	0.2	3.7	0.2	3.8	0.2 [20]
**HDL (mmol/L)**	1.5	0.1	1.5	0.1	1.4	0.1 [20]
**EI (kJ/day)**	8061.1	435.2 [20]	8266.7	440.1 [21]	8023.6	398.8 [19]
**Carbohydrate (%)†**	38.7	1.5 [20]	37.8	1.8 [21]	41.1	1.3 [19]
**Fat (%)†**	35.4	1.3 [20]	35.6	1.3 [21]	35.2	0.1 [19]
**Protein (%)†**	19.8	0.8 [20]	21.3	1.2 [21]	19.3	1.0 [19]
**Alcohol (%)†**	3.0	1.2 [20]	2.4	0.7 [21]	1.4	0.4 [19]
**Fibre (g)**	18.1	1.2 [20]	14.6	1.0 [21]	17.9	1.3 [19]
**EE (kJ/day)**	17089.1	967.1 [17]	16659.7	1052.7 [20]	15911.1	665.9 [19]
**Steps/day**	-	-	8735.1	480.8 [19]	7567.8	793.2 [19]

*Unless indicated by [n]

**°**refers to percentage of total body weight

†refers to percentage of total energy intake; SEM: Standard Error of the Mean; BMI: body mass index; FBG: fasting blood glucose; SBP: systolic blood pressure; DBP: diastolic blood pressure; TC: total cholesterol; TAG: triacylglycerides; LDL: low density lipoprotein; HDL: high density lipoprotein; EI: energy intake; EE: energy expenditure.

### Metabolic syndrome risk factors

The primary outcome measure and a selection of other disease risk factors were collected at four time points following baseline (weeks 6, 12, 18 and 24). The secondary outcome measures were collected at two time points following baseline (weeks 12 and 24), as referred to above (and in Table 1: Schedule of outcome measures.) The paragraphs below summarise the between group differences in changes to baseline measures at each designated time point.

**Fig 2:**
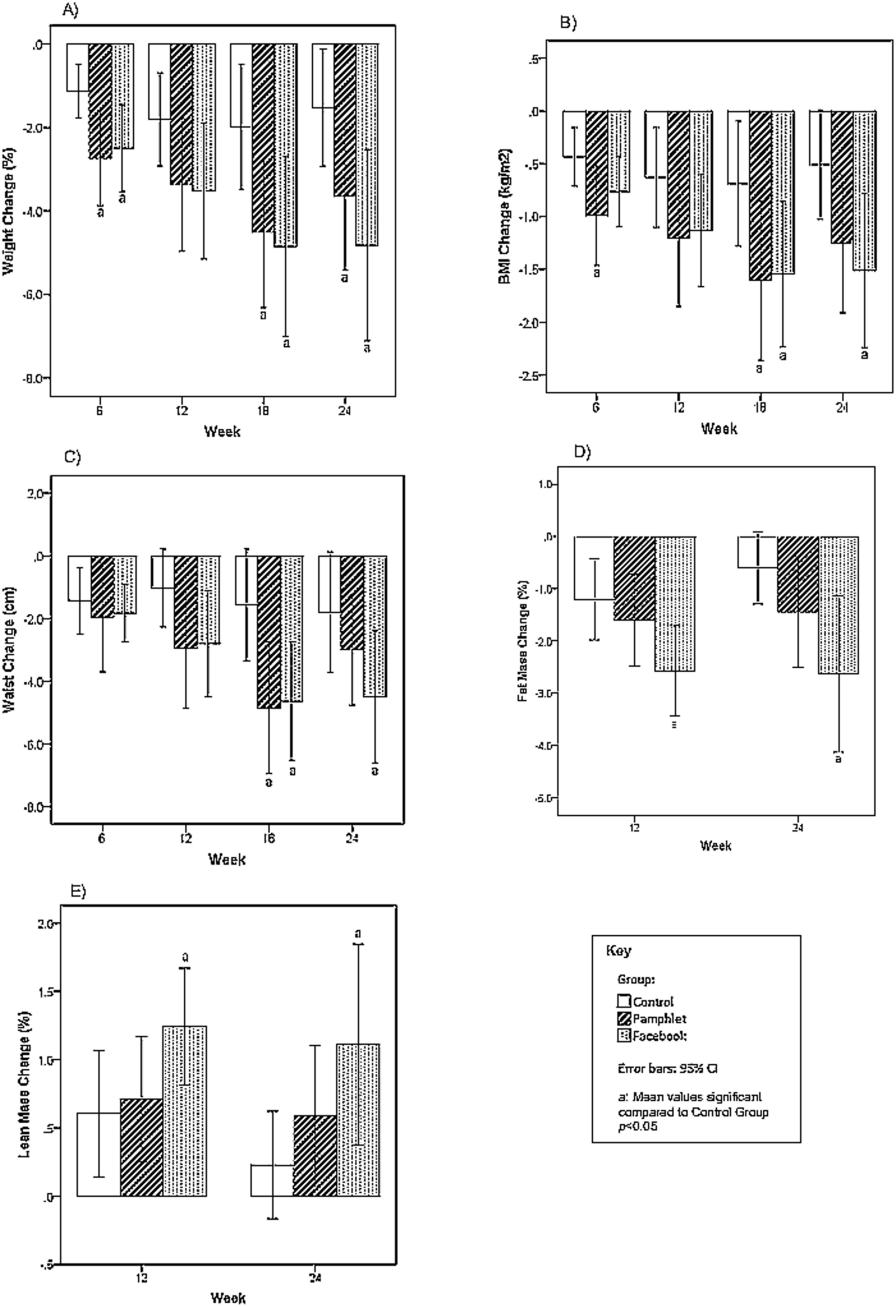
Significant between group differences in outcome measures. (A) weight; (B) BMI; (C) waist; (D) fat mass; (E) lean mass.

The primary outcome measure for this study was change in weight. Both the PG and the FG had significantly greater weight loss than the CG at week 6 (-2.7%, *p* = 0.01 and -2.5%, *p* = 0.02 respectively), at week 18 (-4.5%, *p* = 0.02 and -4.9%, *p* = 0.02 respectively) and at week 24 (-3.6%, *p* = 0.05 and -4.8%, *p* = 0.01 respectively) (Fig 2A). While the FG experienced greater weight loss at weeks 12, 18 and 24 compared to the PG group, these differences were not statistically significant at any time. Compared to the CG, the PG showed a significant reduction in BMI at week 6 (-1.0 kg/m^2^, *p* = 0.03), both the PG and the FG showed significant reductions at week 18 (-1.6 kg/m^2^, *p* = 0.04 and -1.5 kg/m^2^, *p* = 0.04 respectively), but only the FG maintained this change at week 24 (-1.5 kg/m^2^, *p* = 0.02) (Fig 2B).

The PG and the FG experienced statistically significant reductions in waist circumference compared to the CG at week 18 (-4.8 cm, *p* = 0.01 and -4.6 cm, *p* = 0.01 respectively), but only the FG sustained this significant change at week 24 (-4.5 cm, *p* = 0.04) (Fig 2C). There were no significant differences between group reductions in hip measurements across the intervention.

The PG group had significant reductions in fasting blood glucose compared to the CG and the FG. At week 6 a difference of -0.1 mmol/L was statistically significant against the CG (+0.4 mmol/L, *p* = 0.02) and against the FG (+0.4 mmol/L, *p* = 0.007), at week 12 a difference of -0.2 mmol/L was significant against the CG (+0.3 mmol/L, *p* = 0.04) and the FG (+0.4 mmol/L, *p* = 0.001). At week 18 a difference of -0.1 mmol/L was significant against the CG only (+0.6 mmol/L, *p* = 0.04), and at week 24 a difference of -0.4 mmol/L was significant against the CG (+0.4 mmol/L, *p* = 0.04) and the FG (+0.4 mmol/L, *p* = 0.03).

The FG showed numerically greater reductions in fat mass than both the CG and the PG, and was statistically significant reduction compared to CG, at both weeks 12 and 24 (-2.6%, *p* = 0.01) (Fig 2D). Similarly, the FG showed numerically greater increases in lean mass than both the CG and the PG at both times, but this was statistically significant against the CG only, at week 12 (+1.2%, *p* = 0.03) and at week 24 (+1.1%, *p* = 0.03) (Fig 2E).

There were no significant between group differences in blood pressure measurement during the intervention, with the exception of a reduction in systolic blood pressure in the PG compared to the CG at week 6 (-10.3 mmHg, *p* = 0.05) which was not maintained at week 24 (Table 2).

**Table 2:** Between group differences in outcome measures.

	**Week 6**			**Week 12**			**Week 18**			**Week 24**		
	mean	SEM	*n*	mean	SEM	*n*	mean	SEM	*n*	mean	SEM	*n*
Weight (%)*												
○ **Control**	-1.1	0.3	22	-1.8	0.5	18	-2.0	0.7	18	-1.5	0.6	17
○ **Pamphlet**	-2.7^a^	0.5	23	-3.4	0.7	21	-4.5^a^	0.8	19	-3.6^a^	0.8	18
○ **Facebook**	-2.5^a^	0.5	23	-3.5	0.8	22	-4.9^a^	1.0	20	-4.8^a^	1.1	19
**BMI (kg/m^2^)**												
○ **Control**	-0.4	0.1	22	-0.6	0.2	18	-0.7	0.3	18	-0.5	0.2	17
○ **Pamphlet**	-1.0^a^	0.2	23	-1.2	0.3	21	-1.6^a^	0.4	19	-1.3	0.3	18
○ **Facebook**	-0.8	0.2	23	-1.1	0.3	22	-1.5^a^	0.3	20	-1.5^a^	0.4	19
**Waist (cm)**												
○ **Control**	-1.4	0.5	22	-1.0	0.6	18	-1.6	0.8	18	-1.8	0.9	17
○ **Pamphlet**	-2.0	0.8	23	-2.9	0.9	21	-4.8^a^	1.0	19	-3.0	0.8	18
○ **Facebook**	-1.8	0.4	23	-2.8	0.8	22	-4.6^a^	0.9	20	-4.5^a^	1.0	19
**Hip (cm)**												
○ **Control**	-0.3	0.6	22	-1.1	0.6	18	-1.1	0.6	18	-1.5	0.6	17
○ **Pamphlet**	-1.3	0.6	23	-2.5	0.7	21	-2.6	0.7	19	-3.2	0.6	18
○ **Facebook**	-1.3	0.5	23	-2.4	0.7	22	-2.8	0.8	20	-3.3	0.9	19
**FBG (mmol/L)**												
○ **Control**	0.4	0.1	22	0.3	0.2	18	0.6	0.2	18	0.4	0.3	17
○ **Pamphlet**	-0.1^a^^b^	0.2	23	-0.2^a^^b^	0.1	21	0.1^a^	0.2	19	-0.4^a^^b^	0.2	18
○ **Facebook**	0.4	0.1	23	0.4	0.1	22	0.5	0.1	20	0.4	0.3	19
Fat Mass (%)**												
○ **Control**	-	-	-	-1.2	0.4	17	-	-	-	-0.6	0.3	17
○ **Pamphlet**	-	-	-	-1.6	0.4	21	-	-	-	-1.4	0.5	18
○ **Facebook**	-	-	-	-2.6^a^	0.4	22	-	-	-	-2.6^a^	0.7	19
Lean Mass (%)**												
○ **Control**	-	-	-	0.6	0.2	17	-	-	-	0.2	0.2	17
○ **Pamphlet**	-	-	-	0.7	0.2	21	-	-	-	0.6	0.2	18
○ **Facebook**	-	-	-	1.2^a^	0.2	22	-	-	-	1.1^a^	0.3	19
**SBP (mmHg)**												
○ **Control**	-	-	-	-2.8	3.0	17	-	-	-	3.5	2.9	17
○ **Pamphlet**	-	-	-	-10.3^a^	2.2	21	-	-	-	-0.2	2.7	18
○ **Facebook**	-	-	-	-9.6	3.2	22	-	-	-	-3.0	2.0	19
**DBP (mmHg)**												
○ **Control**	-	-	-	-2.1	1.5	17	-	-	-	1.1	1.5	17
○ **Pamphlet**	-	-	-	-4.5	1.3	21	-	-	-	-0.1	1.4	18
○ **Facebook**	-	-	-	-3.4	1.5	22	-	-	-	-0.5	1.0	19
**Insulin (mU/L)**												
○ **Control**	-	-	-	-1.1	0.9	13	-	-	-	0.1	0.7	17
○ **Pamphlet**	-	-	-	-1.3	0.7	19	-	-	-	1.0	0.9	17
○ **Facebook**	-	-	-	-0.9	0.5	17	-	-	-	-0.1	0.9	17
**TC (mmol/L)**												
○ **Control**	-	-	-	-0.3	0.2	12	-	-	-	0.1	0.1	16
○ **Pamphlet**	-	-	-	-0.4	0.2	21	-	-	-	-0.1	0.2	18
○ **Facebook**	-	-	-	-0.3	0.1	17	-	-	-	-0.2	0.1	17
**TAG (mmol/L)**												
○ **Control**	-	-	-	-0.1	0.1	12	-	-	-	0.1	0.2	16
○ **Pamphlet**	-	-	-	-0.1	0.0	21	-	-	-	0.4	0.3	18
○ **Facebook**	-	-	-	-0.2	0.1	17	-	-	-	-0.2	0.1	17
**LDL (mmol/L)**												
○ **Control**	-	-	-	-0.3	0.2	12	-	-	-	0.0	0.1	16
○ **Pamphlet**	-	-	-	-0.3	0.1	21	-	-	-	-0.1	0.1	18
○ **Facebook**	-	-	-	-0.3	0.1	17	-	-	-	-0.2	0.1	17
**HDL (mmol/L)**												
○ **Control**	-	-	-	0.0	0.0	12	-	-	-	0.1	0.0	16
○ **Pamphlet**	-	-	-	-0.1	0.0	21	-	-	-	0.0	0.0	18
○ **Facebook**	-	-	-	0.0	0.0	17	-	-	-	0.0	0.0	17

*percentage of initial body weight

**percentage of total body weight

SEM: Standard Error of the Mean; BMI: body mass index; DBP: diastolic blood pressure; FBG: Fasting blood glucose; HDL: high density lipoprotein; LDL: low density lipoprotein; SBP: systolic blood pressure; TAG: triacylglycerol; TC: total cholesterol

^a^mean values significantly different to Control Group (*p*<0.05)

^b^mean values significantly different to Facebook Group (*p*<0.05).

### Diet and physical activity

According to self-reported food intake data, the differences in mean energy intake between the three groups at all four time points compared to baseline were not found to be statistically significant. The greatest numerical reductions in energy intake was observed in the FG at week 24 (to wit CG: -1107.4 kJ/day v PG: -1071.6 kJ/day v FG: -1465.9 kJ/day). Both the PG and the FG showed numerical reductions in carbohydrate intake at each time point; however, the only significant reduction was in the PG at week 6 compared to the CG (-3.8%, *p* = 0.05).

There were no significant between group differences in either fat or alcohol intake across the intervention.

There were increases in protein intake in the three groups at each time point; however, those increases that were significantly different compared to the CG at week 6 were PG (+5.9%, *p* = 0.05) and the FG (+5.2%, *p* = 0.03), and the FG compared to the CG at week 12 (+4.8%, *p* = 0.05). Notable increases in fibre intake occurred in the PG compared to the FG at week 6 (+2.6 g, *p* = 0.005) and at week 18 (+2.4 g, *p* = 0.03), and in the PG compared to the CG at week 24 (+2.4 g, *p* = 0.03).

**Fig 3:**
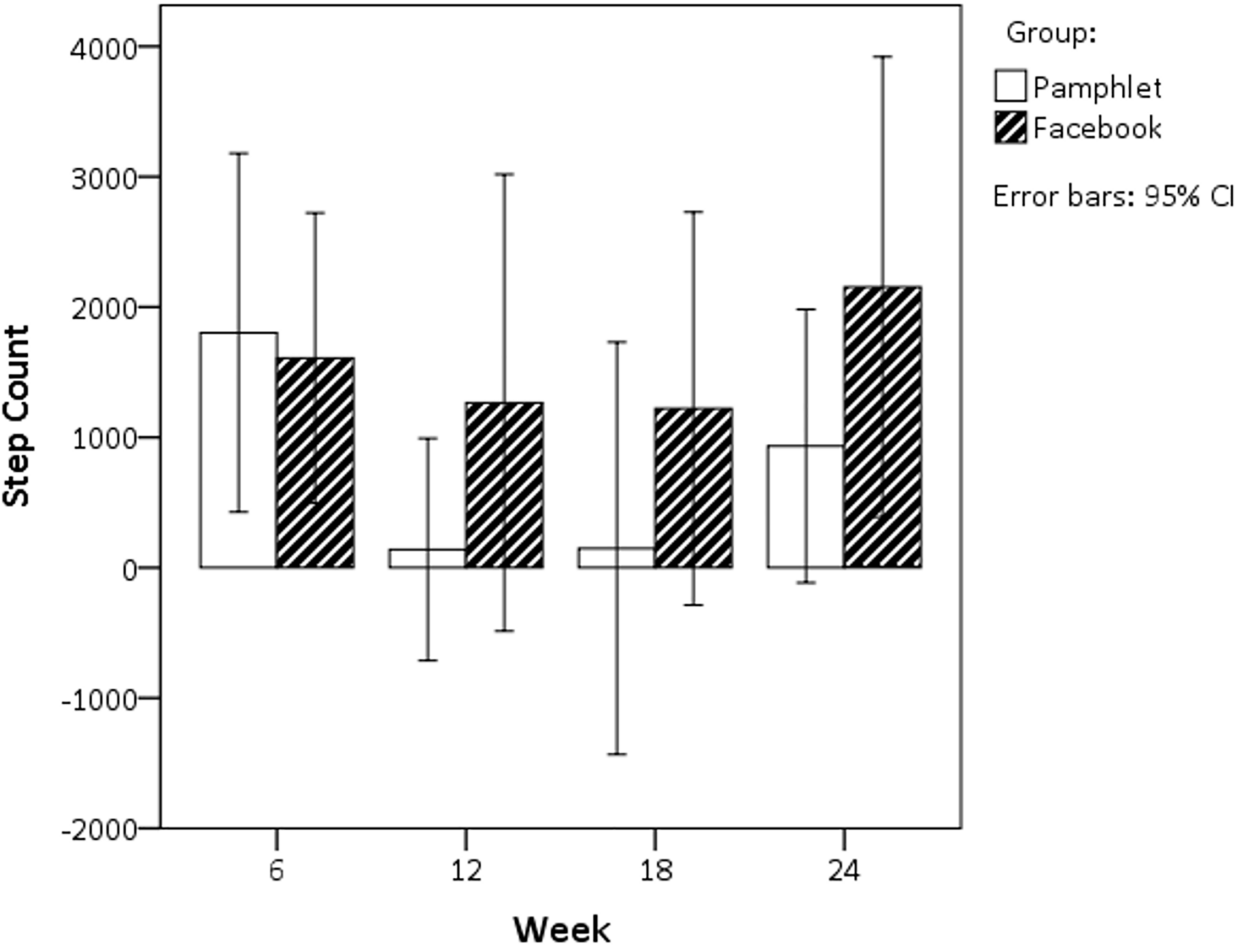
Between group differences in step counts.

The only significant increase in self-reported energy expenditure was recorded in the FG at week 6 (+588.8 kJ/day, *p* = 0.03) compared to the PG. When measuring physical activity, the step counts were not significantly different, although the FG recorded two-fold greater step numerical count compared to PG at the conclusion of the intervention (PG: +933.1 steps v FG: +2153.5 steps) (Fig 3, Table 3).

**Table 3:** Changes to Diet and physical activity.

	**Week 6**			**Week 12**			**Week 18**			**Week 24**		
	mean	SEM	*n*	mean	SEM	*n*	mean	SEM	*n*	mean	SEM	*n*
**EI (kJ/day)**												
○ **Control**	-931.0	468.2	16	-990.5	665.4	15	-1839.9	748.2	11	-1107.4	547.4	15
○ **Pamphlet**	-1843.6	501.0	20	-1693.1	448.0	20	-2320.9	547.2	17	-1071.6	500.3	17
○ **Facebook**	-1498.1	350.1	16	-1539.3	431.5	17	-1570.3	389.7	17	-1465.9	515.3	17
**CHO (%)**												
○ **Control**	0.6	1.5	16	-3.1	2.2	15	-5.0	1.9	11	0.1	2.1	15
○ **Pamphlet**	-3.8^a^	1.5	20	-3.2	1.8	20	-2.2	3.0	17	-3.2	1.7	17
○ **Facebook**	-3.3	1.2	16	-4.3	1.6	17	-5.2	2.1	17	-3.0	1.7	17
**Fat (%)**												
○ **Control**	-1.8	1.4	16	0.5	2.1	15	2.1	2.3	11	-0.9	1.4	15
○ **Pamphlet**	-2.7	1.9	20	-1.8	1.8	20	-2.4	2.8	17	0.0	2.0	17
○ **Facebook**	-1.8	1.2	16	-1.4	1.4	17	1.4	1.7	17	-2.0	1.6	17
**Protein (%)**												
○ **Control**	1.3	1.3	16	0.5	1.2	15	4.7	1.6	11	1.3	1.6	15
○ **Pamphlet**	5.9^a^	1.8	20	4.0	1.5	20	5.2	2.5	17	3.2	1.8	17
○ **Facebook**	5.2^a^	1.1	16	4.8^a^	1.8	17	3.9	1.2	17	4.8	1.9	17
**Alcohol (%)**												
○ **Control**	-0.6	1.2	16	0.6	1.8	15	-2.2	1.9	11	-0.6	0.6	15
○ **Pamphlet**	-0.3	0.6	20	-0.5	0.5	20	-1.4	0.9	17	-0.3	0.6	17
○ **Facebook**	-0.5	0.2	16	0.5	0.4	17	-0.5	0.3	17	-0.5	0.5	17
**Fibre (g)**												
○ **Control**	0.1	1.5	16	-0.9	1.9	15	-2.0	2.1	11	-1.8	1.5	15
○ **Pamphlet**	2.6^c^	1.6	20	1.0	1.2	20	2.4^c^	1.4	17	2.4^a^	1.4	17
○ **Facebook**	-3.2	1.1	16	-1.9	1.4	17	-2.8	1.9	17	-1.7	1.7	17
**EE (kJ/day)**												
○ **Control**	311.7	421.4	15	-836.2	365.2	14	600.6	819.1	9	249.9	808.2	12
○ **Pamphlet**	-855.8	399.2	19	-1046.1	454.2	19	-1472.3	447.6	17	-1626.0	552.0	16
○ **Facebook**	588.8^b^	498.6	17	-142.9	857.1	16	-277.1	452.7	15	-263.8	545.1	16
**Steps/day**												
○ **Control**	-	-	-	-	-	-	-	-	-	-	-	-
○ **Pamphlet**	1802.4	637.5	19	139.7	393.1	18	148.2	724.1	17	933.1	476.0	16
○ **Facebook**	1608.9	510.0	17	1265.6	789.4	15	1221.6	627.4	14	2153.5	795.3	15

%: percentage of energy intake; CHO: carbohydrate; EI: Energy intake; EE: energy expenditure

^a^mean values significantly different to Control Group (*p*<0.05); SEM: Standard Error of the Mean

^b^mean values significantly different to Pamphlet Group (*p*<0.05)

^c^mean values significantly different to Facebook Group (*p*<0.05)

## Discussion

One of the central tenets of health promotion is to create a supportive environment, conducive to health behaviour change [41]. The intervention reported here was designed to provide dietary and physical activity instructions and social support within a dedicated Facebook group, creating a supportive environment for overweight and obese participants to manage their weight.

The overall aim of this study was to determine if a weight management program delivered via a dedicated social media group would augment beneficial changes in weight and other metabolic syndrome risk factors compared to written instructions only in overweight and obese individuals.

It was expected that by week 24 the PG would experience a mean weight loss of 2% of initial body weight compared to CG. As the results show, the PG experienced a mean weight loss of 3.6% of initial body weight, 2.1% greater than the CG (*p* = 0.05). The PG also had greater improvements in fasting blood glucose compared to the CG (*p* = 0.04) and the FG (*p* = 0.03) at the conclusion of the intervention.

It was also expected that, compared to the CG, the FG would experience a mean weight loss of 9% initial body weight at the end of the 24 week intervention. While the FG posted the greatest weight loss by week 24 compared to the CG (*p* = 0.01), at 4.8% of initial body weight, the FG didn’t achieve that predicted target. However compared to the CG, the FG only demonstrated significant improvements in BMI (*p* = 0.02), waist circumference (*p* = 0.04), lean mass (*p* = 0.03) and fat mass (*p* = 0.01) by week 24. Also, although the FG showed greater numerical improvements in weight, BMI, waist circumference, lean and fat mass compared to the PG, these changes were not statistically significant.

By the end of the 24 week trial period, the FG reported the greatest numerical reduction in energy intake (-1465.9 kJ/day) compared to the PG and the CG, though this result did not achieve statistical significance. The discrepancies between weight loss and changes to dietary intake may be explained by inaccurate dietary intake self-reporting, a common problem in weight management trials [7]. The between group differences in baseline energy expenditure measurements recorded for all groups appears to indicate *reductions* in physical activity across the course of the study, with few exceptions; this is inconsistent with the trial recommendations. The between group comparisons show an increase in energy expenditure measurements at week 6 in the CG (+311.7 kJ/day, *p* = 0.05) and the FG (+588.8 kJ/day, *p* = 0.03) compared to the PG, and an increase was noted at week 18 in the CG (+600.6 kJ/day, *p* = 0.03) compared to reductions in the PG and the FG. These results are also inconsistent with the increase in baseline step counts reported in the PG and FG. While the differences were not statistically significant at any of the time points, both groups reported increases in step counts with the PG recording more steps at week 6, but the FG recording greater increases in number of steps for the remaining three time points (including a difference of +1220.4 steps at week 24 compared to the PG).

Overall the changes to weight, BMI, waist, lean and fat mass measures observed in the present study in the FG are very encouraging, particularly in light of the smaller than expected sample size of this study. While these changes were not significant in the present study for the FG compared to the PG, they represent successful, practical outcomes. For example, a 5% reduction in total body weight can provide clinically significant changes to metabolic syndrome risk factors such as lipid profiles [42, 43] and fat mass [42] in overweight/obese individuals. The 4.8% reduction in total body weight and the reductions in BMI, waist circumference, fat mass and energy intake that were noted together with the increases in lean mass and step count posted by the FG are thus very encouraging.

With respect to relevant considerations in interpreting the current findings, it should be noted that the original 12-week intervention (with a 12-week follow-up) was extended to a 24-week intervention only as the week 12 collection point was due to occur during the Christmas and New Year period, and data collected at this time may not have reflected the dietary and physical activity changes made during the twelve weeks prior [30]. It was not advisable to delay the start of the trial by another 12 weeks to avoid the festive season, as many of those individuals recruited at the start of the recruitment period were likely to lose interest in the study if it had been delayed further. According to ‘Stages of Change’ theory, if an individual was at the ‘Preparation Stage’ at recruitment it would be optimal for them to commence the intervention within 30 days; if they were at the ‘Action Stage’, they would need to start the intervention immediately [44, 45]. In addition, it was not feasible to adopt a ‘rolling recruitment’ approach, as it was important for all Facebook Group participants to be given access to the group page at the *same* time, to avoid any social disadvantage within the group. Therefore *all* participants were required to be randomised into groups before the trial commenced. In addition, due to necessity limited resources were spent on recruitment, which may have extended the length of the recruitment phase.

Even so, the effect of this intervention on weight measures and metabolic syndrome risk factors may have been blunted by the occurrence of Christmas in the middle of the intervention period. Excessive food consumption during Christmas is a well-known and common phenomenon [46–49]. The between group differences in outcome measures data (Table 2) shows very few statistically significant results were recorded at this time (week 12). It is speculated that this period may have especially been detrimental to the FG as they may have spent less time online receiving support, information and help, due to the commitments of the season. Alternatively, the results of a clinical weight management trial conducted across such a time period could be viewed as more representative of real world scenarios, as opposed to contriving ideal study conditions that rarely occur in day to day life.

In this trial the weight management guidelines were briefly explained to participants at an initial information session, but beyond that participants were given no further guidance or counselling, as would be the case with most free living individuals making such modifications. One of the reasons for this is that it is common for participants in weight management trials to have the benefit of regular dietetic counseling [50–53] and/or personalised feedback of some kind [54, 55]. In addition, participants in some previous trials had access to food items consistent with the recommended diet [56], which is sometimes provided in dietary meal-sized portions [50], or of specific macronutrient composition [50, 51] and/or provided with kitchen scales [51, 53, 54]. This type of high level of support would require considerable financial expenditure were participants expected to pay, and does not reflect real world scenarios (especially among low socioeconomic groups). Indeed, the results of weight management trials conducted in this way may not represent realistic outcomes for individuals or population groups. However, it is quite possible that social media groups such as the one in the present study may benefit from active leadership from within the group [57] to encourage greater program engagement. This strategy would also maintain cost-effectiveness of the intervention by keeping health professional involvement down to a minimum. Additional facilitator involvement in the FG may be another strategy that could be used to boost participant engagement. It may also be the case that the use of social media for weight management may appeal differentially to certain individual or personality types [58].

Other factors may have influenced the outcomes of this intervention. Ambivalence towards health food choices and/or weight loss has been shown to result in poorer weight loss outcomes, such that an individual with a negative attitude towards the task or their ability to undertake it can undermine the execution of positive intentions [59–61]. Anecdotally provided information in this study indicated that several FG participants accessed a hard copy of the *Total Wellbeing Diet*, which may have meant that they had a reduced need to access the Facebook group page. In addition, one study has shown participants to view social connectedness on Facebook to be distinct from social connectedness with offline connections, i.e. in the ‘real world’ [24]. Perhaps participants in the present study did not rely on each other in the same way that they would typically rely on their offline social connections, particularly as participants were unknown to each other before trial commencement. Furthermore, participants did not choose to join the Facebook Group, but were placed there via study randomisation. Any reluctant social media users within this group may have been less inclined to engage with the other group members online, which could potentially blunt the overall changes to group outcomes.

For many individuals, particularly those in the obese category (BMI ≥30 kg/m^2^), weight loss requires continued effort, not only to maintain a relatively small amount of weight loss, but to persevere until a healthy weight is achieved [62]. Due to the cost-effectiveness of social media, particularly when using existing social media platforms, an ongoing intervention or program delivered within an online social media group may help participants to make sustained progress towards their personal goals. Being a longer-term member of an online group than was possible in the present study may also help to build stronger relationships between members, as stronger online relationships have been shown to improve trust between members [63], and may therefore result in better outcomes over time.

In spite of the issues discussed, the FG reported numerically greater improvements in weight, BMI, waist circumference, fat mass, lean mass, and energy intake compared to the CG and the PG, and a greater step count than the PG, by the end of the 24 week intervention. These results demonstrate the potential of social media to assist overweight and obese individuals with respect to dietary and physical activity modifications for weight management. Further research is needed to clarify these results, and to identify the particular features of social media that may be most beneficial for weight management programs, as well as the types of individuals most likely to benefit from this approach.

### Strengths

The results of this study demonstrate the potential benefits of using social media tools to assist overweight and obese individuals with dietary and physical activity modifications for weight management. As mentioned above, a mean weight loss of 5% of total body weight can result in positive metabolic changes in overweight and obese individuals. In the current study, a mean weight loss of 4.8% of initial body weight was noted in the FG in conjunction with positive changes in waist circumference and in both lean and fat mass. Research in this area is still in its relative infancy, and the results of this trial add significantly to the current knowledge base while suggesting potential benefits that can be applied in the context of both public health and clinical practice.

### Limitations

The results suggest that social media has some potential to assist with weight management, and identifies areas where improvements can be made to optimise this potential. However, the small sample size may have limited the capacity of this study to produce any further statistically significant results. Based on the total sample available at week 24 (*n* = 54) and the observed effect size (Cohen’s *d* = 0.37*)*[38], *post hoc* analysis found that this study achieved a statistical power of 0.65 (or 65%). The statistical methods employed in this study were chosen with generalisability in mind; to wit, in real world settings some individuals may not persevere with a specific weight management program for any length of time e.g. twenty four weeks. Participant burden may have influenced attrition [64] in the present study, as a large amount of data was collected, including psychometric measures and Facebook group activity, which will be analysed and presented in future reports. While the high volume of data collected may have contributed to participant burden, examination of this data may provide further clues to the outcomes reported here.

### Implications for research and practice

Social networking platforms may provide several benefits to group members, such as bridging geographical boundaries, connecting with likeminded individuals (which may be particularly helpful if offline support is lacking), and providing support at low cost and 24-hour accessibility. The ability of group members to assist each other via social media may also remove some of the burden from health care services, for instance between appointments. The potential advantages to health professionals of social networking platforms also include the ability to deliver relatively low cost health interventions, the capacity to manage large caseloads in a time-effective manner and the possibility of reaching minority or hard to access groups. This potential is enhanced if a ready-made platform such as Facebook is used, as this further minimises costs and provide health professionals with access to existing social networks.

Future intervention trials involving social media may benefit from allocating participants to either a ‘treatment program’ or a ‘treatment program with a social media’ group according to their personal preference, as this may reflect more realistically how this resource would be utilised in clinical settings. Participants that are identified as very active social media users could be given leadership roles within these groups to assist with overall participant engagement. Allowing participants to get to know each other a little beforehand (through social media or other channels), or to enroll with one or more friends, may also improve trial outcomes. In addition, future weight management trials may need to accommodate food-related events like Christmas and other relevant time periods (e.g. Passover, Ramadan) in order to inform improved strategies for weight management practices. Information gained from such approaches is likely to help clarify how to make the best use of social media in both the research and the clinical environments.

## Supporting information

S1 File PLOS CONSORT 2010 checklist. (DOC)

S2 File Trial Protocol Part 1. Intervention program.
(PDF)

S3 File Trial Protocol Part 2. Project Outline.
(DOCX)

S4 File Trial Protocol Part 3. Questionnaires.
(DOCX)

S1 Fig Showing up-to-date schedule of outcome measures. (TIF)

## Abbreviations

BMI: Body mass index
CG: Control group
FG: Facebook group
PG: Pamphlet group
