# Supplementary material for: Effects of a weight management program delivered by social media on weight and metabolic syndrome risk factors in overweight and obese adults: A randomised controlled trial
Source: PLoS One. 2017 Jun 2;12(6):e0178326. doi: 10.1371/journal.pone.0178326 (PMC5456050; doi:10.1371/journal.pone.0178326)
Supplement: S2 File — Intervention program. (PDF) [file pone.0178326.s002.pdf]

The CSIRO Total Wellbeing Diet

By Dr Manny Noakes

with Dr Peter Clifton

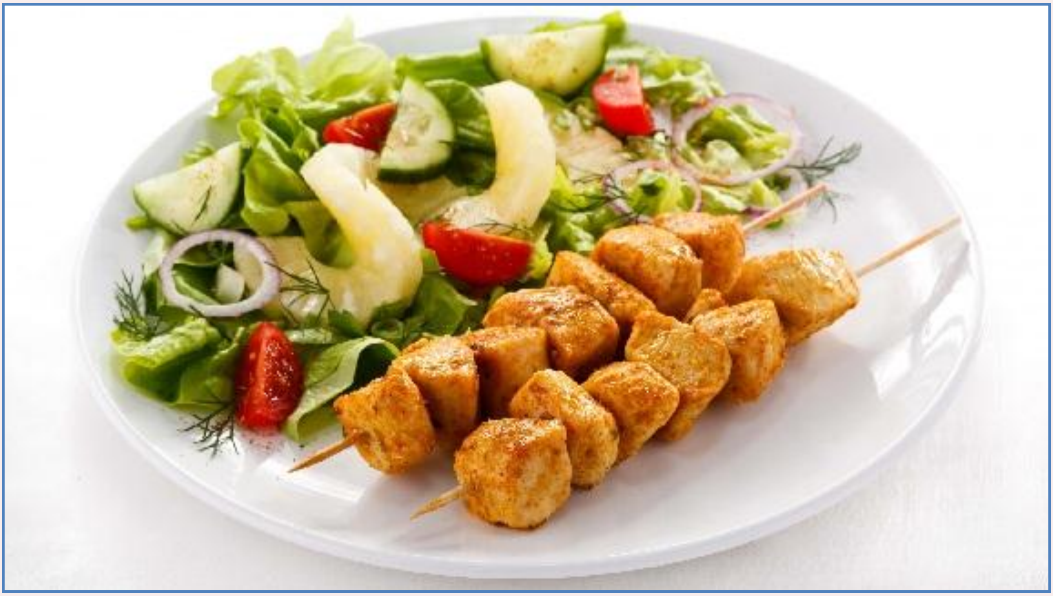

This booklet contains excerpts from The CSIRO Total Wellbeing Diet Book 2, and The CSIRO Total Wellbeing Diet Recipes on a Budget, (digital editions published in 2013), and was compiled by Monica Jane. Reproduced with kind permission from Penguin Australia Pty Ltd.

The material contained within this booklet is subject to copyright and is not to be reproduced without written permission from Penguin Australia Pty Ltd.

The CSIRO Total Wellbeing Diet

Our studies, and those of overseas researchers, have shown that a higher-protein, low-fat diet helps:

- preserve muscle during weight-loss
- enhance loss of fat
- improve vitamin B12 and iron status, and
- lower triglyceride levels in the blood.

In our studies, those people on a higher-protein diet lost the same amount of weight as those on a higher-carbohydrate diet, since the two diets offered an equal amount of kilojoules and the same amount of fat. However, body composition (that is, the ratio of fat to muscle) showed greater improvement among those people on the higher-protein diet. When the participants in other studies were allowed to eat until they were no longer hungry, those on the higher-protein diet lost more weight than those on the higher-carbohydrate diet, even after more than a year.

The reduction in hunger and the beneficial effect on muscle provided by the higher-protein diet is mostly related to its protein content, while the reduced triglyceride levels and enhanced fat-loss seem to be related to its lower amounts of carbohydrate. The diet is healthy because its protein comes from lean red meat, fish, chicken and low-fat dairy products, all of which provide good nutrition. A high-protein diet in which the protein comes from protein powders and supplements is unlikely to be healthy, unless the supplements are fortified with vitamins and minerals.

The basic plan ...

LEAN PROTEIN FOODS

– 2 units a day for dinner

1 unit is equal to 100 g raw weight of protein food, including red meat, chicken or fish (without bones). Eat red meat 3 times a week for dinner. Eat fish at least twice a week for dinner.

– up to 1 unit a day for lunch

Eat up to 100 g (raw weight) of any lean protein source (tinned or fresh fish or seafood, chicken, turkey, red meat or 2 eggs) each day for lunch. Eat red meat up to once a week for lunch.

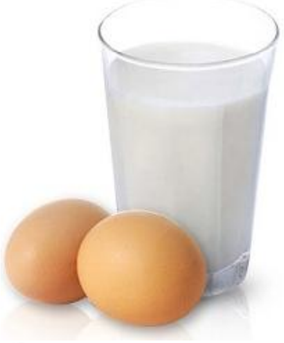

DAIRY

– 3 units a day

1 unit is equal to:

- 250 ml reduced-fat milk
- 200 g reduced-fat or diet yoghurt
- 200 g reduced-fat custard or dairy dessert
- 25 g cheddar cheese or other full-fat cheese
- 50 g reduced-fat cheese (less than 10 per cent fat)

Please ignore any page numbers referred to in the text of this booklet as they are incorrect.

WHOLEGRAIN BREAD

– 2 units a day

1 unit is equal to one 35 g slice. You can replace 1 unit each day with any of the following:

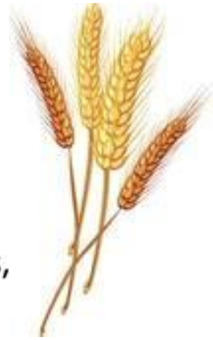

- 1 slice fruit loaf
- 2 crispbread, such as Ryvita
- 1 medium potato (about 150 g)
- 4 tablespoons cooked rice or noodles
- ½ cup (about 50 g) cooked pasta
- 4 tablespoons baked beans, or cooked lentils, kidney beans or other legumes
- 40 g high-fibre cereal or rolled oats

VEGETABLES

– at least 2½ units a day from free list

1 unit is equal to 1 cup (80–150 g) cooked vegetables. See free list (right) for vegetables you can eat. We recommend ½ unit salad and 2 cups (160–300 g) cooked vegetables each day.

FRUIT

– 2 units a day

1 unit is equal to 150 g fresh or tinned unsweetened fruit, 150 ml unsweetened fruit juice or 30 g dried fruit.

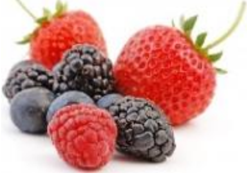

HIGH-FIBRE CEREAL

– **1 unit a day**

1 unit is equal to:

- 40 g any high-fibre breakfast cereal (e.g. Sultana Bran, Fibre Plus)
- 1 Weet-Bix plus ½ cup (35 g) All-Bran
- 40 g rolled oats
- 1 slice wholegrain toast

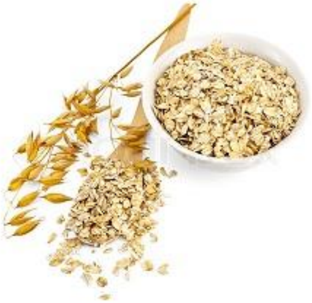

FATS AND OILS

– **3 units added oils or fats a day**

1 unit is equal to 1 teaspoon any liquid oil such as canola, olive or sunflower oil. 3 units oil is equal to:

- 3 teaspoons soft (trans-fat-free) margarine
- 6 teaspoons light margarine
- 3 teaspoons curry paste in canola oil
- 60 g avocado
- 20 g nuts or seeds

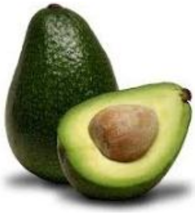

INDULGENCE FOODS

– **up to 2 units a week**

As a general rule, 1 unit is equal to any food or drink providing approximately 450 kJ, such as 150 ml wine or 20 g chocolate.

The free list: anytime foods and drinks

These foods contain minimal kilojoules, so eat them freely to spice up your meals.

vegetables artichokes, asparagus, bean sprouts, beetroot, bok choy, broccoli, Brussels sprouts, cabbage, capsicum, carrots, cauliflower, celery, chilli, chives, choko, corn, cucumber, eggplant, fennel, green beans, lettuce, marrow, mushrooms, onion, parsnips, peas, pumpkin, radishes, rhubarb, silver beet, spinach, swedes, tomatoes, turnip, zucchini

drinks Bonox, Bovril, cocoa, coffee, diet cordial, diet soft drinks, herbal tea, tea, unflavoured mineral water, water

condiments artificial sweeteners, barbecue sauce, chilli sauce, clear soup, curry powder, diet jelly, diet topping, fish sauce, garlic, ginger, herbs, hoisin sauce, horseradish, lemon, mint sauce, mustard, oil-free salad dressing or mayonnaise, parsley, pickles, soy sauce, spices, stock cubes, tomato paste, tomato sauce, Vegemite, verjuice, vinegar, wasabi

Note: It is acceptable to use small amounts of cornflour, custard powder or sugar to thicken or sweeten dishes. 1 level teaspoon cornflour, custard powder or sugar has 40–60 kJ. This is low enough not to worry about if you use them only occasionally. Always use salt sparingly.

DAILY UNITS OF EACH FOOD TYPE

low-fat dairy	3 units
lean protein	3 units (dinner 2, lunch 1)
high-fibre cereal/bread	3 units
healthy fats	3 units
vegetables/salad	at least 2½ units
fruit	2 units
indulgences	2 units a week

DAILY UNIT BREAKDOWN

3 units protein	red meat contains protein, well-absorbed iron, zinc, vitamin B12; fish contains protein, omega-3 fatty acids; chicken contains protein, zinc
1 unit cereal, 2 units bread	contains fibre, slow-release carbohydrate, B vitamins, magnesium
3 units dairy	contains calcium, protein, vitamin B12, zinc
2½ units vegetables, 2 units fruit	contains folate, vitamins A, B6 and C, fibre, magnesium, antioxidants
3 units fats	oils and margarines contain vitamin E; margarine also contains vitamins A and D

Sample meal plan for one day

Breakfast

40 g high-fibre cereal (e.g. Fibre Plus) with
250 ml low-fat milk and 150 g fruit
Breakfast = 1 unit cereal (see page 8),
1 unit dairy, 1 unit fruit

Lunch

2 slices wholegrain bread with 1 teaspoon margarine,
up to 100 g chicken, fish, pork, lamb, beef, or 2 eggs,
and ½ cup salad vegetables
Lunch = 1 unit protein, 2 units bread,
½ unit vegetables, 1 unit fats

Dinner

200 g (raw weight) beef, lamb, pork, chicken or fish
cooked in 2 teaspoons oil, with at least 2 cups vegetables
(from free list, see page 9)
150 g fruit with 200 g low-fat dairy dessert
Dinner = 2 units protein, 1 unit dairy, 1 unit fruit,
2 units vegetables, 2 units fats

Snack options

tea or coffee with low-fat milk
200 g low-fat yoghurt
Snack = 1 unit dairy

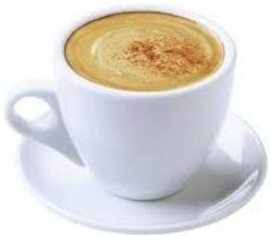

FOR MEN

As men tend to be taller and heavier than women, it may be necessary to add extra food units to the daily allowance. The following list contains examples of items that can be added. As a general rule, add up to 2 items per day. Each item listed adds 500 kJ to your daily energy intake.

low-fat milk	250 ml
wholegrain bread	1 x 35 g slice
fresh fruit salad	300 g
dry-roasted, unsalted almonds	20 g
potato crisps cooked in canola oil	1 x 21 g packet
avocado	¼
baked potato	150 g
cooked pasta	⅔ cup
lean beef, lamb, pork, chicken or fish	100 g
canned beans	140 g
cheese	30 g
cheesecake	75 g
oil	3 teaspoons
ice-cream	70 g
milk chocolate	25 g
wine	150 ml glass
beer	375 ml can
spirits	60 ml

alcohol and wellbeing

For many people, drinking alcohol is a way of winding down at the end of a busy working day, and it's true that moderate amounts of alcohol can act as a relaxant. Many people have commented that the alcohol restrictions in the CSIRO Total Wellbeing Diet are difficult to maintain. But there are several reasons why it's important to keep your alcohol intake very low when you are trying to lose weight. First of all, alcohol is a significant source of kilojoules: 1 g of alcohol provides 27 kJ, which adds up to 414 kJ in a standard 150 ml glass of wine, 550 kJ in a 375 ml can of beer and even more in mixed drinks with added fruit juice or soft drinks. These high-kilojoule drinks do not satisfy our appetite at all, nor add any essential micronutrients to our diet. In addition, because alcohol stimulates the biochemical pathways involved in appetite control, too much alcohol also means you can end up overeating. Thirdly, alcohol can slow down fat metabolism, making it even harder to lose weight.

what is a standard drink?

In Australia, a standard drink contains 10 g alcohol, which is the equivalent of 12.5 ml pure alcohol. It does not indicate a standard volume, since different drinks can have widely different alcohol contents. By law, the label must indicate how many standard drinks are in that container. The table below will help a little, but always check the label.

You do not need to become a teetotaller to lose weight, nor should you take up drinking alcohol if you don't already. But what is a safe and sensible approach to losing weight on the CSIRO Total Wellbeing Diet if you really enjoy having a drink more often than once or twice a week? If you do not want to lose weight, a safe intake of alcohol is 2 standard drinks a day for women and 4 for men (see more information overleaf). (Remember, an average glass of wine is more like 1.5 standard drinks – see table overleaf.) If you are trying to lose weight on the Diet, however, an alcohol intake of 1 standard drink a day will slow your weight-loss only slightly. If this works for you, great, but if you find that it sabotages your eating pattern, try 3 standard drinks a week.

STANDARD DRINKS AND KILOJOULE COUNTS

wine	white, non-sweet	150 ml glass	414	1.5
	white, medium sweet	150 ml glass	414	1.5
	white, sparkling	150 ml glass	407	1.5
	red, still	150 ml glass	425	2.0
	red, sparkling	150 ml glass	407	2.0
beer	bitter/draught	375 ml can	550	1.5
	reduced alcohol (1.15–3.5% alcohol)	375 ml can	393	0.8
	stout	375 ml can	855	2.0
spirits	non-sweet	60 ml	514	2.0
port	standard	60 ml	374	0.8
liqueur	cream-based, coffee-flavoured	60 ml	867	1.0
	other (greater than 30% alcohol)	60 ml	1084	2.0

Useful tips...

- Will I be able to feed my whole family with the suggested meals?

Yes, you will. The recipes fit easily into family meal plans. However, members of the family who do not need to lose weight may need to include extra carbohydrate foods in their meals, such as bread, pasta, rice or potatoes.

The diet is adequate for overweight children and teenagers from a nutritional perspective. However, the number of kilojoules will need to be adjusted for the age, size and activity level of the child, which is best done by a qualified dietitian. Because children are growing, excessive kilojoule restriction can affect their growth, so some care needs to be taken to ensure that their diet is not overly restrictive. We would recommend smaller weight-losses each week than for adults, unless the child is very overweight. Sometimes even keeping their weight stable as they grow will result in fat-loss. If the whole family is eating meals based on the CSIRO Total Wellbeing Diet, this will provide good nutrition for everyone. Extra snacks (mostly fruit and low-fat dairy snacks) may be necessary for some overweight children. We recommend you consult your GP and seek a referral to a dietitian, who will keep an eye on your child's weight and growth.

- Can I eat sardines instead of tuna or salmon?

You can eat 100 g lean protein (fish, chicken, pork, ham, lamb or turkey) for lunch each day, so sardines are perfectly acceptable, and are a fantastic source of omega-3 fatty acids and calcium. If the sardines are packed in oil, count this as part of your fat intake, unless you drain them.

- Can I eat eggs?

As eggs are basically a protein source, you can eat them instead of other protein foods. Substitute 1 egg for 50 g lean meat, chicken, turkey, pork, ham or fish.

- What can I eat for snacks?

Here are some alternative morning- and afternoon-tea ideas: from your dairy allowance a skim-milk cappuccino or latte, or a 200 g low-fat yoghurt, custard or Frûche; or from your fruit allowance 1 piece (150 g) fruit. Tea or coffee with a dash of milk is fine. A low-kilojoule soup as part of your vegetable allowance is a great option as well.

- How much sugar or honey can I have? Can I have artificial sweeteners?

Although sugar is generally not included in the Diet, 1–2 teaspoons a day is fine. Artificial sweeteners will not affect your kilojoule intake, so they will not ruin your diet.

1 Take it easy with the alcohol

- Alcohol contains lots of kilojoules and may also add kilojoules by increasing the amount you eat (see page 17).
- Alternate your alcoholic drinks with mineral water or a diet soft drink.
- Don't allow top-ups of your alcoholic drinks. They make it too easy to lose track of how much you're drinking!

2 Watch the nibbles

- If you're going to a friend's house for dinner or having friends over, be careful with the nibbles – they can be kilojoule kryptonite!
- Use vegetables rather than biscuits for dipping. Sticks of carrot, celery and cucumber, or mushroom caps, snowpeas, parboiled cauliflower and broccoli, all look fabulously colourful and appetising. Dips such as carrot and coriander, hummus, tzatziki, eggplant or beetroot taste great and are light on kilojoules.
- Include fruit as nibbles. Cherries, strawberries and slices of melon are great alternatives to the deep-fried morsels often served.
- Try to avoid salty chips, and biscuits and pastries. If serving nuts, leave them in their shells so that you can't eat them as quickly.
- Eat slowly and savour the taste. Spend more time talking, listening and laughing.

Three things to consider when eating out…

3 Count your courses

- It's helpful not to be over-hungry when you go out to dinner. A good idea is to eat one of your dairy units, such as a tub of yoghurt, an hour or so before going out. That should take the edge off your hunger for a while.
- If you're having more than one course, try to choose a soup as an entrée – vegetable-based soups are best. Remember to ask the chef not to add cream.
- Dishes consisting mainly of protein foods, such as smoked salmon, carpaccio, or chicken or prawn dishes, can be a good option if the meat has not been battered and deep-fried.
- If there are no suitable mains, a safe bet is to choose 2 entrées instead of an entrée and a main.
- Avoid hoeing into the bread and butter.
- If there is a set menu, don't eat everything on your plate. Eat all of the protein-food component and the free list vegetables, but only half the mash or chips.
- Restaurants never seem to serve main meals with enough vegetables these days. Always ask for a side salad or steamed vegetables on the side. They will slow down your eating and fill you up as well.
- Desserts are always tricky. If you can resist, great! If not, it's best to share one.

Sample recipes

Breakfast

bircher muesli with bran

Serves 8 (makes 4 cups)

1 cup rolled oats

1 cup untoasted muesli

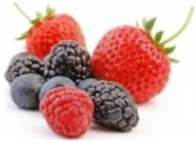

1 cup unprocessed wheatbran

2 cups hot water

2 tablespoons lemon juice

¼ cup almonds

200 g low-fat flavoured yoghurt

1 large green apple, grated

150 g seasonal fruit (berries, peaches, nectarines, bananas) per serve

2 tablespoons honey

Place the oats, muesli and bran in a large ceramic dish and pour water and lemon juice over. Allow to soak for 30 minutes. Add almonds, yoghurt and apple and stir to combine thoroughly. Cover and refrigerate overnight.

Serve with fruit and a drizzle of honey, plus extra yoghurt from your dairy allowance, if desired.

1 serve of ½ cup muesli with fruit = 1 unit cereal,
2 units fruit

leftovers

Bircher muesli keeps for – and actually improves over – about four days. Make this recipe on Sunday night and enjoy a nutritious and easy breakfast well into the week.

Breakfast smoothies

SERVES 2 PREP **5 mins**

A complete breakfast in itself, a smoothie is a quick and easy way to get the day started with minimal fuss. This one combines rockmelon and banana – see below for two more delicious flavour combinations.

2 cups (450 g) chopped rockmelon
1 ripe banana
1¼ cups (310 ml) reduced-fat milk, reduced-fat soy milk or reduced-fat almond milk
125 g reduced-fat plain yoghurt
1 tablespoon honey
4 tablespoons bran flakes
pinch ground cinnamon
small handful ice cubes

1 SERVE =
½ unit bread
1 unit dairy
1 unit fruit

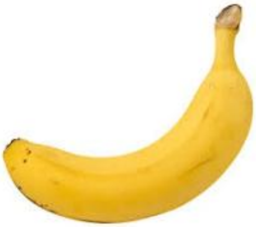

1 Place all the ingredients into a blender and blend until smooth.

Variations

MANGO AND COCONUT: Replace the rockmelon with the flesh from one mango. Use ⅔ cup (160 ml) reduced-fat coconut-flavoured evaporated milk and ½ cup (125 ml) water in place of the milk.

MIXED BERRY: Replace the fruit with 2 cups (300 g) mixed fresh or frozen berries, and use reduced-fat strawberry yoghurt instead of plain. Omit the cinnamon.

1 SERVE (1 SLICE) =	½ unit fruit
2 units bread	¾ unit fats

Breakfast banana bread

MAKES 8 SLICES PREP **15 mins** COOK **1 hour**

This bread is especially good lightly toasted. Freeze leftover slices, individually wrapped in plastic wrap, for up to 2 weeks.

cooking oil spray
1¼ cups (200 g) wholemeal self-raising flour
¾ cup (110 g) self-raising flour
½ cup (12.5 g) powdered sweetener
1 teaspoon ground nutmeg
1 teaspoon baking powder
½ cup (125 ml) reduced-fat buttermilk or reduced-fat milk
1 egg, lightly beaten
1 teaspoon vanilla extract or essence
3 ripe bananas, mashed (you'll need 1½ cups/360 g mashed banana)
4 tablespoons chopped walnuts or pecans

1 Preheat the oven to 180°C. Spray a 21 cm × 9 cm loaf tin with cooking oil and line with baking paper.

2 Combine the flours, sweetener, nutmeg and baking powder in a large bowl and mix well. In another bowl, whisk the buttermilk or milk, egg and vanilla together. Make a well in the dry ingredients and pour in the milk mixture. Stir in the mashed banana and chopped nuts.

3 Spoon the batter into the prepared tin and smooth the top. Bake for 55–60 minutes until lightly golden and a skewer inserted in the centre comes out clean. If the top is browning too much, cover with foil for the last 5 minutes of cooking.

4 Leave to cool in the tin for 10 minutes before turning out onto a wire rack to cool. Allow to cool to room temperature before slicing.

5 Store in an airtight container for up to 3 days.

Soft boiled eggs with cheesy soldiers and baked mushrooms

Serves 2 Prep 10 mins

Cook 15 mins

1 SERVE =
1 unit protein
1 unit bread
1 unit dairy
1 unit vegetables
½ unit fats

4 small–medium (about 150 g) field mushrooms, stems removed and finely chopped, mushrooms left whole

1½ teaspoons light margarine

½ teaspoon thyme leaves or finely grated lemon zest

1 tablespoon balsamic vinegar

4 eggs, at room temperature

2 slices wholemeal bread

50 g tasty cheese, grated

Tabasco sauce, to serve

1 Preheat the oven to 180°C, and line a baking tray with baking paper.

2 In a small bowl, combine the chopped mushroom stems with the margarine and thyme leaves or lemon zest. Place the mushrooms, cap-side down, on the baking tray and dot with the margarine mixture, then drizzle with balsamic vinegar and season to taste. Bake for 12–15 minutes until tender.

3 Meanwhile, shortly before the mushrooms are ready, bring a small saucepan of water to the boil. Add the eggs to the boiling water and cook for 4 minutes for a runny yolk (cook for a minute longer if you prefer a set yolk). Remove and place in egg cups.

4 Lightly toast the bread, then sprinkle over the cheese and drizzle with a few drops of Tabasco. Place under a hot grill until melted and golden, then carefully cut each slice into 2 cm thick soldiers.

Spiced ricotta fruit toast

SERVES 4 PREP **5 mins** COOK **2 mins**

The combination of ricotta cheese, cinnamon and honey needs only toasted fruit bread to make it sing. Use a sourdough fruit bread if you have it.

120 g fresh reduced-fat ricotta
½ teaspoon ground cinnamon
1½ tablespoons honey
4 slices sourdough fruit bread
1 mango, peeled and stone removed, flesh cut into cubes or 2 bananas, peeled and sliced
4 tablespoons fresh or frozen blueberries
½ teaspoon ground nutmeg, optional

1. Place the ricotta, cinnamon and 2 teaspoons of the honey in a bowl and mix until smooth.

2. Toast the bread and spread with the ricotta mixture. Arrange the fruit and blueberries on top and sprinkle with nutmeg, if using. Drizzle over remaining honey just before serving.

Tip: Use any in-season fruit you like: try strawberries, peaches, nectarines or apricots.

**1 SERVE =**	**½ unit dairy**
**1 unit bread**	**½ unit fruit**

Lunch

spiced red lentil & vegetable soup

LUNCH serves 4

2 teaspoons olive oil
1 carrot, roughly chopped
1 onion, roughly chopped
2 sticks celery, roughly chopped
1 clove garlic, crushed
1 tablespoon freshly grated ginger
1 cup dried red lentils
2 teaspoons garam masala
½ teaspoon chilli powder
1 × 400 g tin tomatoes
1 litre water
freshly ground black pepper
⅓ cup chopped coriander (cilantro)
⅓ cup chopped flat-leaf (Italian) parsley
⅓ cup low-fat natural yoghurt

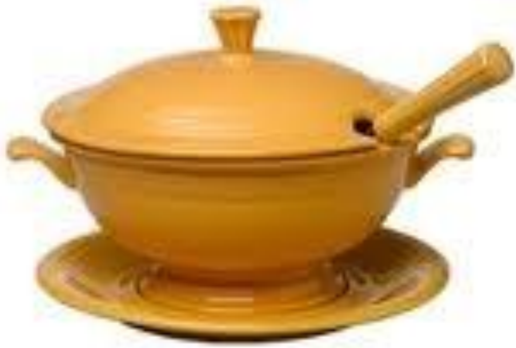

Heat oil in a heavy-based saucepan over medium heat. Add carrot, onion and celery and cook for 5 minutes, or until vegetables are soft. Add garlic, ginger, lentils and spices and stir to combine. Add tomatoes and water and bring to a boil. Reduce heat and simmer for 30 minutes, or until lentils are soft. Season with pepper and stir through coriander and parsley. Spoon into bowls and serve with a dollop of yoghurt.

1 serve = 2 units bread, 1 unit vegetables, ½ unit fats

warm zucchini and green bean salad with lemon and sumac

Serves 4 Prep 15 minutes

Cook 15 minutes

Tip: For 1/2 a protein unit add 2 soft-boiled eggs to each serve

1 SERVE =
2 units vegetables
1 unit fats

2 lemons, peeled and pith removed
3 corn cobs
4 zucchinis (courgettes), cut on the diagonal into 3 cm thick slices
1 carrot, sliced into 1 cm rounds
75 g green beans, trimmed
80 g baby spinach leaves
large handful flat-leaf parsley or mint leaves

DRESSING
1 tablespoon olive oil
1 clove garlic, crushed
1–2 teaspoons ground sumac
2 tablespoons white balsamic vinegar

1 Segment the lemons by holding them over a bowl and using a small, sharp knife to cut the flesh away from the inner membrane, letting the juice drop into the bowl. Slice each segment in half lengthways, then add to the bowl and set aside.

2 For the dressing, combine all the ingredients and set aside to allow the flavours to infuse.

3 Bring a large saucepan of water to the boil, add the corn and cook for 10 minutes until tender. Remove with tongs then, when cool enough to handle, place the cobs on their ends and carefully slice off the kernels.

4 Steam the zucchini, carrot and green beans for 5 minutes until tender, adding the corn for the last minute to reheat. Transfer the vegetables and spinach to a large bowl, drizzle over the dressing and season to taste with salt and pepper. Toss through the lemon segments and parsley or mint and serve.

Ham and cheese wraps with wholegrain mustard and pickles

SERVES 4 PREP **10 mins**

Pickled vegetables add pizazz to the classic ham and cheese wrap. This wrap tastes great toasted too – just cook in a sandwich press for 2 minutes until golden on both sides.

4 wholemeal mountain breads
4 tablespoons wholegrain mustard or English mustard
400 g lean, salt-reduced ham
1 carrot, coarsely grated
2 tomatoes, sliced
1 cup (150 g) Pickled Zucchini or Pickled Cucumber **(see page 201)**
¼ red (Spanish) onion, thinly sliced
120 g reduced-fat tasty cheese, grated

1 Spread each piece of mountain bread with 1 tablespoon mustard. Arrange the ham and vegetables on top, then sprinkle over the cheese and roll to enclose.

**1 SERVE =**	**¾ unit dairy**
**1 unit protein**	**1¼ units vegetables**
**1 unit bread**	

lemon & chilli chicken skewers

LUNCH serves 4

1 clove garlic, crushed

¼ cup lemon juice

1 green chilli, seeded and finely chopped

½ cup buttermilk

400 g skinless chicken breast, cut into 2 cm cubes

In a large bowl, combine garlic, lemon juice, chilli and buttermilk. Add chicken and turn to coat thoroughly. Cover bowl and allow chicken to marinate for 30 minutes.

If using bamboo skewers, soak them in hot water for 30 minutes before use.

Preheat a grill plate or barbecue grill to high.

Thread chicken pieces onto 8 skewers. Grill for 2 minutes each side – 8 minutes in total – or until cooked through.

Serve skewers with a large helping of your favourite salad or steamed vegetables.

1 serve = 1 unit protein

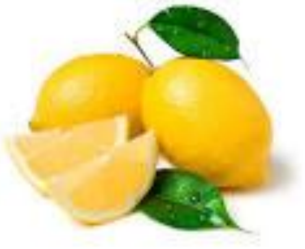

rosemary lamb with olive & feta salad

LUNCH serves 4

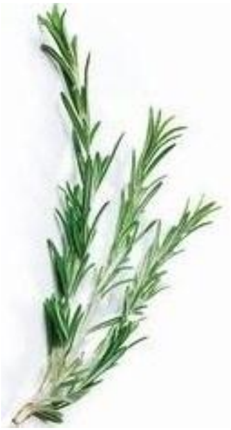

1 tablespoon chopped rosemary

1 clove garlic, crushed

3 teaspoons redcurrant jelly

400 g lamb fillets, trimmed of fat and sinew

1 tablespoon olive oil

12 green olives

100 g low-fat feta

2 baby cos lettuces, outer leaves discarded, leaves separated

1 bulb fennel, finely sliced

1½ tablespoons oil-free balsamic dressing

In a small bowl, mix rosemary, garlic and redcurrant jelly. Add lamb and toss to coat thoroughly.

Heat oil in a large non-stick frying pan over medium heat. Add lamb and cook for 3 minutes each side, or until done to your liking. Remove lamb from heat, cover with foil and set aside to rest for 5 minutes.

In a large bowl, combine olives, feta, cos leaves and fennel. Toss with balsamic dressing and divide among serving plates. Slice lamb thickly on the diagonal and arrange on top of salad.

1 serve = 1 unit protein, ½ unit dairy, 1½ units vegetables, 1 unit fats

chicken, tomato & zucchini pizza

LUNCH serves 4

4 small wholemeal pita breads
⅓ cup tomato paste
2 teaspoons dried Greek oregano
2 ripe tomatoes, thinly sliced
2 zucchini (courgettes), thinly sliced lengthways
400 g cooked skinless chicken breast, sliced
100 g feta or mozzarella
1⅓ cups rocket (arugula)
1 tablespoon olive oil

Preheat oven to 200°C (390°F).

Spread pita breads with tomato paste and sprinkle with oregano. Divide tomato and zucchini between pitas, add a layer of sliced chicken and crumble feta or mozzarella over the top. Bake for 10 minutes.

Serve topped with rocket leaves and a drizzle of olive oil and with a salad alongside.

1 serve = 1 unit protein, 2 units bread, 1 unit dairy, ½ unit vegetables, 1 unit fats

pita bread pizzas

Pizzas are a delicious way to use up leftovers. Keep a packet of pita breads in the freezer for emergency meals, and use any toppings you like.

Mediterranean mini muffins

Makes 24 Prep 15 minute

Cook 25 minutes , plus cooling time

cooking oil spray
1 tablespoon olive oil
½ small onion, finely chopped
4 tablespoons finely chopped red capsicum (pepper)
1 teaspoon dried Italian or mixed herbs
4 tablespoons wholemeal self-raising flour
4 tablespoons self-raising flour
½ teaspoon smoked paprika
4 tablespoons reduced-fat milk
50 g reduced-fat tasty cheese, grated
1 egg, beaten
2 tablespoons chopped black olives
1 tablespoon salt-reduced tomato paste (puree)

1 SERVE (2 MINI MUFFINS) =
¼ unit bread
¼ unit vegetables
¼ unit fats

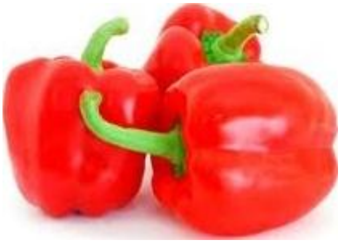

Tomato and onion salad

SERVES 4 PREP **10 mins, plus standing time**

½ small red (Spanish) onion, halved, thinly sliced
1 tablespoon extra virgin olive oil
1 clove garlic, crushed
1 tablespoon sherry vinegar or red wine vinegar
600 g tomatoes, sliced into 5 mm thick rounds
½ teaspoon smoked paprika

1 SERVE =
2 units vegetables
1 unit fats

1 Preheat the oven to 200°C and spray a 24-hole mini-muffin pan with cooking oil (or line with paper cases).

2 Place a frying pan over medium heat and add the olive oil, onion, capsicum and dried herbs. Cook, stirring, for 7–8 minutes until the vegetables are soft. Transfer the mixture to a large bowl and leave to cool for 5 minutes.

3 Sift the flours into a medium-sized bowl, then add the paprika and a pinch of salt and pepper and combine.

4 Stir the milk, cheese, egg, olives and tomato paste into the onion mixture. Make a well in the centre of the dry ingredients, then add the wet ingredients and mix until just combined. Spoon the batter into the muffin holes, filling each to two-thirds full.

5 Bake the muffins for 15–17 minutes until lightly browned, then leave to cool in the pan for 5 minutes before turning out onto a wire rack. Serve the muffins warm or at room temperature.

---

1 Place the onion in a bowl and cover with cold water. Set aside for 15 minutes, then drain well.

2 Combine the olive oil, garlic and vinegar and mix well.

3 Arrange the tomato slices on a large plate, sprinkle with the smoked paprika and season with salt and pepper. Arrange the onion slices on top of the tomato, then drizzle with the dressing. Leave to stand at room temperature for 30 minutes to allow the flavours to develop.

tomatoes stuffed with tuna, basil & spinach

LUNCH serves 4

8 medium-sized ripe tomatoes

400 g tinned tuna in spring water, drained and flaked

½ red (Spanish) onion, finely diced

⅓ cup shredded basil

1 × 250 g packet frozen spinach, defrosted

1 tablespoon finely grated lemon zest

200 g low-fat ricotta

freshly ground black pepper

2 tablespoons olive oil

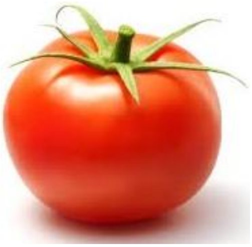

Preheat oven to 170°C (340°F).

Slice tops off tomatoes and carefully remove flesh with a teaspoon, reserving lids and flesh for later. Place tomato shells upside down on paper towels to drain.

Place tuna, onion, basil, spinach, lemon zest, ricotta and tomato flesh in a bowl, season well with pepper and combine thoroughly. Spoon tuna mixture into tomato shells and replace tops. Transfer tomatoes to an ovenproof dish and drizzle with olive oil. Bake for 20 minutes, or until heated through. Serve with roasted zucchini or rocket salad.

1 serve = 1 unit protein, 1 unit dairy,
1 unit vegetables, 2 units fats

lemon tuna patties

LUNCH serves 4 (makes 8 patties)

300 g tinned tuna, drained

1 small red (Spanish) onion, finely chopped

2 tablespoons chopped coriander (cilantro)

2 teaspoons finely grated lemon zest

2 eggs, separated

2 tablespoons wholemeal plain flour

2 tablespoons vegetable oil

Place tuna, onion, coriander and lemon zest in a bowl and mix lightly with a fork. Add egg yolks (reserving whites for later) and continue to mix. Season lightly. Sift flour into mixture, and gently fold through.

Place the egg whites in a clean bowl and whisk until soft peaks form. Spoon the whites into the tuna mixture and gently fold through. With slightly wet hands, form the mixture into 8 patties.

Heat oil in a large non-stick frying pan over medium heat. Add patties and cook for 5 minutes each side, or until golden brown. Drain on paper towels. Serve tuna patties with a mixed-leaf salad tossed with an oil-free dressing.

1 serve = 1 unit protein, 2 units fats

Dinner

beef stroganoff

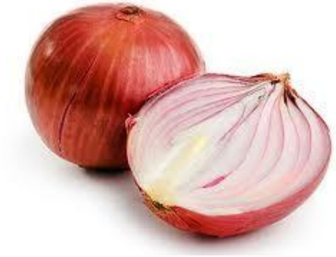

DINNER serves 4

1 tablespoon vegetable oil
800 g lean beef strips
1 large onion, finely sliced
1 clove garlic, crushed
400 g button mushrooms, sliced
¾ cup salt-reduced beef stock
1 tablespoon Worcestershire sauce
1 tablespoon cornflour (cornstarch) mixed with 2 tablespoons cold water
100 g low-fat natural yoghurt
¼ cup roughly chopped flat-leaf (Italian) parsley

Heat oil in a large non-stick frying pan over medium heat. Add beef strips in batches and cook for 5 minutes, or until browned. Return all meat to pan, add onion, garlic and mushrooms and cook for 10 minutes, or until vegetables are soft. Stir in stock, Worcestershire sauce and cornflour mixture and bring to a boil. Reduce heat and simmer, covered, for 10 minutes. Stir in yoghurt and parsley, and season to taste.

Serve with rice or pasta from your daily bread allowance and steamed vegetables.

1 serve = 2 units protein, 1 unit vegetables, 1 unit fats

roasted chicken with thyme, red onions & butternut pumpkin

DINNER serves 4

finely grated zest and juice of 1 lemon
1 clove garlic, roughly chopped
1 tablespoon picked lemon thyme leaves
¼ cup chopped flat-leaf (Italian) parsley
2 tablespoons olive oil
1.5 kg skinless chicken drumsticks and
thighs on the bone, trimmed of fat
4 red (Spanish) onions, peeled and halved
600 g butternut pumpkin, peeled and cut into 4 cm pieces

Preheat oven to 180°C (350°F).

Place lemon zest, lemon juice, garlic, thyme, parsley and half the oil in a food processor and blend until smooth. Grease a large ovenproof baking dish with the remaining oil, arrange chicken pieces in the dish and spoon on herb mixture. Rub mixture into chicken pieces and season lightly. Add onions and pumpkin to dish and cover with foil. Roast for 20 minutes. Remove foil, baste chicken with cooking juices, and bake for a further 40 minutes.

Serve with steamed greens or a salad.

1 serve = 2 units protein, 1½ units vegetables, 2 units fats

coq au vin

DINNER serves 4

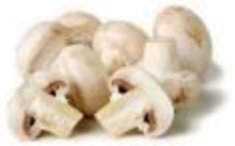

2 tablespoons olive oil
100 g lean bacon, sliced
350 g small brown onions, peeled
1 clove garlic, crushed
900 g skinless chicken drumsticks and thighs on the bone, trimmed of fat
2 cups red wine
1 cup chicken stock
3 Roma (plum) tomatoes, diced
2 bay leaves
1 tablespoon cornflour (cornstarch) mixed with 2 tablespoons cold water
250 g button mushrooms
200 g green beans, trimmed
2 tablespoons chopped flat-leaf (Italian) parsley

Heat half the oil in a large heavy-based saucepan over high heat. Add bacon, onion and garlic and cook for 5 minutes, or until onion is soft. Remove from pan and set aside. Return pan to heat, add remaining oil and cook chicken in batches for 5 minutes, or until golden. Return all chicken and bacon mixture to pan, add wine, stock, tomatoes and bay leaves and bring to a boil. Reduce heat and simmer, covered, for 45 minutes, or until chicken is tender. Remove lid, stir in cornflour mixture and mushrooms and simmer, uncovered, for a further 10 minutes.

Meanwhile, steam beans in a steamer for 5 minutes. Serve coq au vin sprinkled with parsley and with beans on the side.

1 serve = 2 units protein, 1½ units vegetables, 2 units fats

beef & vegetable pasta bake

DINNER serves 4

2 tablespoons olive oil

800 g lean minced (ground) beef

1 red (Spanish) onion, diced

2 teaspoons dried mixed herbs

1 red capsicum (pepper), seeded and finely diced

1 × 400 g tin chopped tomatoes

1 cup salt-reduced beef stock

250 g rigatoni, cooked

2 zucchini (courgettes), diced

1 cup frozen peas

100 g grated low-fat cheddar cheese

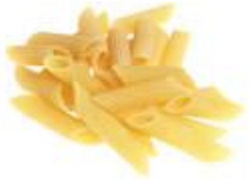

Heat half the oil in a large heavy-based saucepan over high heat. Add mince in two batches and cook for 5 minutes, or until just browned. Transfer to a large bowl. Add remaining oil to pan, along with onion, and cook for 5 minutes, or until onion is soft. Return mince to pan, add dried herbs, capsicum, tomatoes and stock and bring to a boil. Reduce heat and simmer for 35 minutes. Stir in pasta, zucchini and peas, then carefully pour into a 2 litre ovenproof dish.

Meanwhile, preheat oven to 200°C (390°F). Sprinkle cheese over pasta bake, transfer to oven and cook for 10 minutes, or until cheese is golden. Serve with a mixed-leaf salad.

1 serve = 2 units protein, 1 unit bread, ½ unit dairy, 1 unit vegetables, 2 units fats

Garlic and sage chicken with squash and feta salad

Serves 4 Prep 15 minutes Cook 4 1/2 hours

800 g skinless chicken thighs, trimmed of fat
2 teaspoons sweet paprika
cooking oil spray
1 tablespoon olive oil
1 large leek, trimmed, washed and cut into 1 cm thick rounds
1 teaspoon fennel seeds
2 teaspoons dried sage
3 cloves garlic, crushed
300 g potato, cut into 3 cm pieces
finely grated zest of 1 lemon
1½ cups (375 ml) Chicken Stock

SQUASH AND FETA SALAD

cooking oil spray
6 yellow patty pan squash (about 240 g), cut into 1 cm thick slices
2 zucchinis (courgettes), cut into 1 cm thick rounds
80 g feta
2 teaspoons drained capers
juice of 1 lemon

1 Dust the chicken with 1 teaspoon of the paprika. Spray a large frying pan with cooking oil and heat over medium–high heat. Working in batches if necessary, cook the chicken thighs for 3–4 minutes until browned all over. Remove and set aside.

2 Reduce the heat to medium and add the olive oil, leek, fennel seeds and sage. Cook for 5–6 minutes until the leek begins to soften. Add the garlic and cook for 2 minutes, then transfer this mixture to the slow-cooker, along with the potato, lemon zest, chicken stock and the remaining paprika. Season to taste with salt and pepper and stir to combine. Place the browned chicken pieces on top, then cover and cook on low for 4–4½ hours until the chicken is tender and the potato is cooked.

3 Meanwhile, for the salad, spray a chargrill pan with cooking oil and heat over medium–high heat. Cook the squash and zucchini for 5–6 minutes, turning regularly, until tender. Place in a large bowl, along with the feta, capers and lemon juice. Season to taste with salt and pepper and toss to combine.

→

4 To serve, divide the leek and potato among four plates and top with the chicken. Spoon over some sauce and serve with the squash and feta salad.

1 SERVE =
2 units protein
½ unit bread
½ unit dairy
2 units vegetables
1½ units fats

Garlic and sage chicken pie

Serves 4 Prep 15 minutes Cook 30 minutes

1 quantity Garlic and Sage Chicken
1 carrot, chopped into 5 mm pieces
1 cup (120 g) frozen peas
finely grated zest and juice of 1 lemon
4 sheets filo pastry
cooking oil spray

1 SERVE =
2 units protein
1 unit bread
2 units vegetables
1 unit fats

1 Preheat the oven to 180°C.

2 Reheat the chicken mixture in a covered saucepan over low–medium heat. Using a slotted spoon, transfer the chicken to a plate, remove the meat from the bone and chop the meat into small chunks. Mash the potato in the saucepan to a chunky mash, then return the chicken to the pan.

3 Steam or boil the carrots for 3–4 minutes until tender, then drain and add to the chicken mixture, along with the peas, lemon zest and 2 tablespoons of the lemon juice. Season to taste with salt and pepper and mix gently to combine, being careful not to break up the chicken.

4 Spray a 1.5 litre ovenproof dish with cooking oil, then spoon the chicken mixture into the dish. Lay out the filo sheets on a clean work surface and spray with cooking oil. Arrange the filo sheets on top of the chicken, then place the dish in the oven and bake for 20–25 minutes until the pastry is golden and crisp.

steamed bream with lemon & capers

DINNER serves 4

1 small red (Spanish) onion, finely diced
1 tablespoon capers, chopped
2 tablespoons flat-leaf (Italian) parsley
2 tablespoons olive oil
juice and finely grated zest of 2 lemons
4 × 200 g bream fillets
300 g baby squash
150 g baby spinach

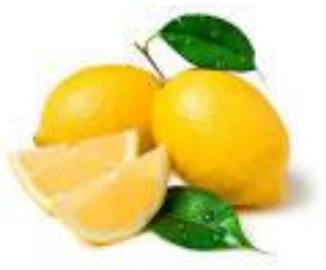

Preheat oven to 200°C (390°F).

In a small bowl, combine onion, capers, parsley, 1 tablespoon of the oil and half the lemon juice and zest.

Tear off 4 large pieces of foil. Place a fish fillet in the centre of each and spoon over lemon and caper mixture. Bring together the long sides of each foil parcel and fold the edge over several times. Now fold in the short ends of the foil several times to ensure the parcel is well sealed. Transfer parcels to a baking tray and cook for 10 minutes, or until fish is cooked through. (The exact cooking time will depend on the thickness of the fillets.)

Meanwhile, steam squash for 5 minutes, or until cooked. Drain and slice thickly while still hot. Transfer to a large bowl and add spinach leaves. Toss to combine, and drizzle with remaining lemon juice and zest. Season lightly and serve alongside the steamed bream.

1 serve = 2 units protein, 1½ units vegetables, 2 units fats

fish stew with tomato & basil

DINNER serves 4

1 tablespoon olive oil

1 leek, white part only, finely sliced and washed

2 sticks celery, sliced

2 cloves garlic, crushed

1 bulb fennel, finely sliced

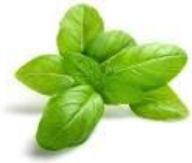

1 × 400 g tin chopped tomatoes

1 cup white wine

2 cups fish stock or chicken stock

800 g fish fillets (blue-eye, flathead or ling), cut into 4 cm pieces

¼ cup roughly chopped basil

finely grated zest of 1 lemon and 2 tablespoons juice

Heat oil in a heavy-based saucepan over medium heat. Add leek, celery, garlic and fennel and cook for 10 minutes, or until vegetables are soft. Add tomatoes, wine and stock and bring to a boil. Reduce heat and simmer for 20 minutes. Add fish and simmer for a further 10 minutes. Gently stir through basil, lemon zest and lemon juice, reserving a little basil and zest to garnish, if desired. Season to taste.

Serve with a big green salad and crusty bread from your daily bread allowance.

1 serve = 2 units protein, 1 unit vegetables, 1 unit fats

pork loin with tomato & sage

DINNER serves 4

1 × 800 g pork loin

16 sage leaves

1 tablespoon olive oil

2 tablespoons lemon juice

3 large ripe tomatoes, cut into 1 cm dice

½ cup white wine

200 g broccolini

200 g carrots

2 tablespoons flat-leaf (Italian) parsley leaves

Cut pork loin into 8 steaks. Place steaks, cut-side up, on a chopping board and cover with plastic wrap. Using a rolling pin, lightly flatten to 5 mm thickness. Remove plastic wrap and place 4 sage leaves on half of the steaks, then top with the remaining steaks. Secure with a toothpick at either end.

Heat oil in a heavy-based frying pan over medium heat. Add pork and cook for 5 minutes each side, or until browned. Remove from pan, cover lightly with foil and set aside to rest.

Return pan to heat, add lemon juice, tomatoes and wine and bring to a boil. Reduce heat and simmer for 2 minutes. Return pork to pan and continue to simmer for 8 minutes.

Meanwhile, steam broccolini and carrots. Divide vegetables among serving plates and arrange pork alongside. Sprinkle pork with parsley and serve immediately.

1 serve = 2 units protein, 1½ units vegetables, 1 unit fats

poached blue-eye with peperonata & basil

DINNER serves 4

1 serve = 2 units protein, 1 unit vegetables, 2 units fats

3 lemons
1 litre chicken stock or fish stock
2 sprigs flat-leaf (Italian) parsley
5 black peppercorns
1 bay leaf
4 × 200 g fillets blue-eye trevalla

PEPERONATA
2 tablespoons olive oil
2 red capsicums (peppers), seeded and sliced
1 red (Spanish) onion, finely chopped
2 cloves garlic, finely chopped
1 cup torn basil
freshly ground black pepper

Cut 1 lemon into slices and place in a deep frying pan along with stock, parsley, peppercorns and bay leaf, and bring to a boil. Reduce heat and simmer for 3 minutes. Add fish and simmer very gently for 8 minutes. Remove pan from heat and allow fish to rest in the liquid.

Meanwhile, heat a large saucepan over medium heat. Add oil and capsicum and cook for 5 minutes, or until the capsicum starts to soften. Add onion and garlic and cook for a further 5 minutes. Stir in basil and season well with pepper. Spoon this peperonata onto serving plates and top with fish. Serve with lemon wedges and your favourite steamed greens.

Zingy lemon chicken

SERVES 4 PREP **15 mins** COOK **25 mins**

A fresh approach to sweet and sour chicken, this dish is zesty, fragrant and packed with flavour. Serve this with any steamed vegetables you like: try carrot, cauliflower, broccoli, snowpeas or green beans.

- 800 g skinless chicken thigh fillets, trimmed of fat, halved
- 3 teaspoons ground turmeric
- cooking oil spray
- 1 tablespoon peanut or vegetable oil
- 2 teaspoons yellow mustard seeds
- 1 red (Spanish) onion, finely diced
- 2 long red chillies, finely sliced
- 1½ tablespoons finely chopped ginger
- 1 cinnamon stick
- 5 cloves
- 1¼ cups (310 ml) Chicken Stock (see page 209)
- finely grated zest of 1 lemon
- juice of 2 lemons
- 2 tablespoons sultanas
- 2 bunches Chinese broccoli (gai lan), trimmed and cut into 3 cm lengths

1 SERVE =
2 units protein
¼ unit fruit
1 unit vegetables
1 unit fats

1 Dust the chicken pieces all over with 2 teaspoons of the turmeric. Place a large frying pan over medium–high heat and spray with cooking oil. Working in batches, cook the chicken for 2–3 minutes until browned on all sides. Remove the chicken and set aside.

2 In the same pan, add the peanut or vegetable oil and mustard seeds and cook until the seeds begin to pop, then add the onion, chilli and ginger and cook for 2 minutes until fragrant. Add the cinnamon stick, cloves and remaining turmeric and cook for 1 minute more. Add the stock, lemon zest and juice and the sultanas and bring to the boil. Season to taste with salt and pepper, then return the chicken to the pan. Stir to combine, then reduce the heat to low and simmer, covered, for 10–12 minutes until the chicken is cooked through. Transfer the chicken to a plate with a slotted spoon and cover to keep warm. Increase the heat to high and boil the sauce, uncovered, for 2–3 minutes until thickened and reduced.

3 Meanwhile, steam or boil the Chinese broccoli for 3–4 minutes until just tender, then drain.

4 To serve, divide the Chinese broccoli among four plates, top with the chicken and spoon over the sauce.

Tip: For some added sweetness, add 1 finely chopped red capsicum along with the spices and cook for 2 minutes. You could use fish or prawns instead of chicken (just adjust the cooking time accordingly).

italian lamb meatloaf

DINNER serves 4

700 g lean minced (ground) lamb

1 onion, finely diced

2 cloves garlic, finely chopped

1 teaspoon dried mixed herbs

3 Roma (plum) tomatoes, finely diced

2 tablespoons tomato sauce (ketchup)

¼ cup roughly chopped flat-leaf (Italian) parsley

70 g wholemeal breadcrumbs

2 eggs, lightly beaten

1 × 250 g packet frozen spinach, defrosted

¼ cup pine nuts, lightly toasted

Preheat oven to 180°C (350°F). Lightly grease a loaf tin.

In a large bowl, mix all ingredients using your hands. Season lightly. Press mixture into loaf tin, cover with foil and bake for 40 minutes. Remove foil and bake for a further 15 minutes, or until a brown crust forms. Tilt loaf tin and drain off any excess liquid, then allow meatloaf to stand for 10 minutes before cutting and serving.

1 serve = 2 units protein, ½ unit bread, 1 unit vegetables, 1½ units fats

pumpkin mash with cabbage & spring onions

SIDES serves 4

1 serve = 2 units vegetables, 2 units fats

2 tablespoons olive oil
500 g cabbage, finely shredded
600 g butternut pumpkin, peeled and cut into large chunks
1 cup low-fat milk
4 spring onions (scallions), finely sliced

Heat olive oil in a large frying pan over medium heat. Add cabbage and sauté for 5 minutes, or until soft. Remove from heat and set aside.

Fill a large saucepan with 2 cm lightly salted water and bring to a simmer. Add pumpkin, cover tightly, and cook for 15 minutes, or until tender. Drain well and return to pan. Add milk and mash pumpkin with a fork. To serve, season mash lightly and stir through cabbage and spring onions.

Goes well with beef or lamb.

cauliflower with leeks & parmesan

SIDES serves 4

1 serve = 1 unit vegetables, ½ unit dairy, 1 unit fats

1 tablespoon olive oil
1 leek, white part only, finely sliced and washed
3 cups small cauliflower florets
½ teaspoon nutmeg
2 cups low-fat milk
¼ cup grated parmesan

Heat oil in a large heavy-based saucepan over medium heat. Add leek and sauté for 5 minutes, or until soft. Add cauliflower, nutmeg and milk and bring to a boil. Reduce heat and simmer, covered, for 8 minutes, or until cauliflower is tender. Season lightly.

Heat grill to high. Drain cauliflower and leek mix and transfer to an ovenproof dish. Sprinkle with parmesan and place under grill until cheese is golden.

Goes well with beef, lamb or chicken.

Desserts

almond jelly with orange segments

Serves 6

450 ml boiling water
7 teaspoons powdered gelatine
375 ml light evaporated milk
1 teaspoon almond essence
2 teaspoons Equal or other powdered sweetener
6 oranges, segmented
¼ cup mint leaves

1 serve = ½ unit dairy, 1 unit fruit

Pour water into a jug, add gelatine and whisk with a fork to dissolve. Add evaporated milk, almond essence and Equal, and mix well. Pour into 6 small ramekins, or other moulds, and refrigerate for 2 hours, or until set.

Before serving, toss orange segments with mint. Serve jellies either in the ramekins or turned out onto plates, and with orange segments.

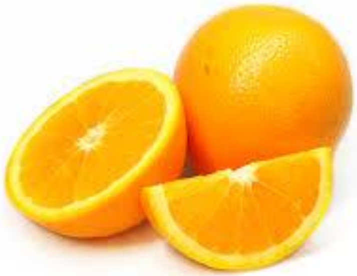

baked apples with cinnamon & ricotta

Serves 4

2 large green apples
⅓ cup rolled oats
¼ cup mixed dried fruit
½ teaspoon ground cinnamon
½ cup low-fat ricotta
2 tablespoons maple syrup
400 g low-fat vanilla yoghurt

Preheat oven to 200°C (390°F).

Peel, core and halve apples and place, cut-side up, on a baking tray lined with baking paper. In a bowl, mix oats, dried fruit, cinnamon and ricotta. Spoon mixture into the core of each apple half, making a small mound. Drizzle with maple syrup and bake for 20 minutes. Serve warm with vanilla yoghurt.

1 serve = 1 unit dairy, 1 unit fruit

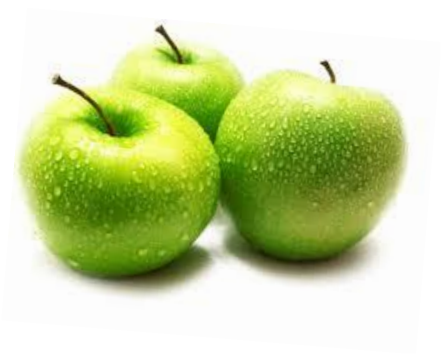

Mango and passionfruit frozen yogurt pops

Makes 8 Prep 10 minutes Freeze 6 hours

These will keep for up to two weeks in the freezer

2 cups (400 g) diced mango
2 cups (560 g) reduced-fat passionfruit yoghurt
3 tablespoons powdered sweetener
2 tablespoons lemon juice (optional)

1 SERVE =
¼ unit dairy
½ unit fruit

1 Place the mango in the bowl of a food processor and process until smooth. Add the yoghurt and sweetener and process until combined. Taste and add lemon juice if desired.

2 Pour into eight popsicle moulds and insert the sticks. Freeze for 6 hours or overnight.

Tip: If you don't have popsicle moulds, pour the mixture into small paper cups, cover with plastic wrap and poke an ice-cream stick through into the yoghurt. To eat, simply remove the plastic wrap and peel away the paper cup.

Variations

RASPBERRY AND CHOCOLATE: replace the mango with 2 cups (300 g) frozen raspberries, use reduced-fat chocolate yoghurt and omit the lemon juice.

BANANA AND HONEY: replace the mango with 2 cups (300 g) chopped ripe banana and use reduced-fat vanilla yoghurt. Add 2 tablespoons of honey instead of the sweetener, and omit the lemon juice if you like.

MIXED BERRY: replace the mango with 2 cups (300 g) frozen mixed berries and use reduced-fat strawberry yoghurt.
