# Supplementary material for: Effects of a weight management program delivered by social media on weight and metabolic syndrome risk factors in overweight and obese adults: A randomised controlled trial
Source: PLoS One. 2017 Jun 2;12(6):e0178326. doi: 10.1371/journal.pone.0178326 (PMC5456050; doi:10.1371/journal.pone.0178326)
Supplement: S3 File — Project Outline. (DOCX) [file pone.0178326.s003.docx]

**Using New Technologies To Promote Weight Management**

**Trial Protocol - Part 1**

Approved by the Curtin University Human Research Ethics Committee

(approval number: HR90/2014)

By:

Monica Jane

Sebely Pal

Martin Hagger

Jonathan Foster

Suleen Ho

Faculty of Health Sciences

Curtin University, Bentley WA

**BRIEF RATIONALE**

Over the last three decades, overweight and obesity and the associated health consequences have become global public health priorities. So far, methods that have been tried and tested to address this problem have not had the desired impact, suggesting quite clearly that other approaches need to be considered. One of the many lessons learned throughout these attempts is that permanent weight loss requires sustained dietary and lifestyle changes, yet adherence to weight management programs has often been noted as one of the biggest challenges. This project aims to address this issue by examining whether the use of social media, as a health promotion tool, will help to improve adherence to a weight management program. To test the effectiveness of this measure, the designated program will be delivered via popular social networking site Facebook, and compared to a standard delivery method that provides exactly the same content but which is communicated through a pamphlet, over a period of twenty four weeks. Although weight loss is expected, this study will primarily investigate the effectiveness of social media as a program delivery method. Therefore the program utilised will be one that has already been proven to achieve weight loss, and will consist of the CSIRO Total Wellbeing Diet, which also incorporates the physical activity guidelines promoted by the Australian Government. One hundred and twenty participants will be recruited from the Perth community, and evenly randomised into either the Facebook group, the pamphlet group, or a control group, the latter following their usual diet and lifestyle. Change in weight will be an initial indicator of adherence to the program. Also monitored will be blood lipids, blood insulin, blood glucose, blood pressure, waist circumference, physical activity, eating behaviour, mental well-being (stress, anxiety, and depression), social support, self-control, self-efficacy, Facebook activity, and Facebook evaluation, as secondary outcome measures. It is expected that this study will support the use of social media as a successful delivery method of a weight management program, as a source of social support and information sharing, thereby enhancing the improvements expected from the dietary and lifestyle changes promoted in this study. Facebook is a popular, easy to access and cost-effective online platform that can be used to assist the formation of social groups, and could be translated into health promotion practice relatively easily. It is anticipated that the results of this project will add to the body of knowledge regarding the use of social media as a vehicle for health promotion interventions, and that social media will provide an invaluable resource for health professionals and patients alike in the near future.

**AIM**

The aim of this project is to measure weight loss and other outcome measures in overweight and obese individuals when a weight management program is delivered via social media, compared to the same program presented in written information only, over a period of twenty four weeks.

**RESEARCH QUESTIONS**

- To determine whether incorporating social media into a weight management program will assist overweight and obese individuals to achieve greater improvements in weight loss and other outcome measures than following the same dietary and lifestyle recommendations in written form alone.
- To elucidate the particular aspects of social media that assist overweight and obese individuals to achieve the greater improvements in weight loss and other outcome measures.

**HYPOTHESES**

In the present study, it is hypothesised that:

- Compared to the Control Group, the Pamphlet Group will experience moderate improvements in metabolic syndrome risk factors, including weight loss of 2% of initial body weight, over the 24 week intervention period.
- Compared to the Control Group, the Facebook Group will experience greater improvements in metabolic syndrome risk factor, including weight loss of 9% of initial body weight, over the 24 week intervention period.
- Participants in the Facebook Group will experience greater improvements in metabolic syndrome risk factors compared to the Pamphlet Group due to the support they receive from using Facebook.

**RESEARCH METHODS**

**Participants:** A cohort of 120 overweight and obese individuals with a body mass index (BMI) between 25-40 kg/m2 and aged between 21 and 65 years, will be recruited from the Perth community via advertisements in the West Australian Newspaper and Community Newspapers. Eligible participants will also be required to have access to a computer, laptop, tablet or Smartphone. Exclusion criteria will include smoking, lipid lowering medication, use of steroids and other agents that may influence lipid metabolism, use of warfarin, diabetes mellitus, hypo- and hyperthyroidism, cardiovascular events within the last 6 months, major systemic diseases, gastrointestinal problems, proteinuria, liver disease, renal failure, weight fluctuations over the past 6 months, vegetarianism and participation in any other clinical trials within the last 6 months. This study will be conducted according to the ethical guidelines laid down in the Curtin University Human Research Ethics Committee and the National Health and Medical Research Council (of Australia).

**Study Design:** This will be a three-armed, randomised, controlled, parallel design intervention study undertaken over a 24-week trial period. Interested participants will be screened and those who meet the selection criteria will be allocated to one of the three groups of 40 participants by block randomisation according to age and gender: the Control Group who will be given the Australian Dietary Guidelines and the National Physical Activity Guidelines for Adults (both easily accessible via the internet); the Pamphlet Group who will be instructed in the weight management program by written information (intervention group 1), and the social media group who will receive the weight management program via Facebook in the Facebook Group (intervention group 2). The twenty four-week weight management program will be presented to the two intervention groups as a condensed version of the CSIRO Total Wellbeing Diet, which includes detailed information of the weight management program and how to follow it, available via Penguin Publishing. The booklet will be compiled from excerpts from both the CSIRO Total Wellbeing Diet Book 2, and CSIRO Total Wellbeing Diet Recipes on a Budget, with permission from Penguin Publishing, thus providing useful and up-to-date information - see Trial Protocol Part 2.) Before the commencement of the trial, participants will attend an information session where details of the study will be explained: the Control Group will be given the Australian Guide to Healthy Eating and the National Physical Activity Guidelines for Adults as standard care, and will receive all materials available to the intervention groups at the completion of the study; the Pamphlet Group will be given the CSIRO Total Wellbeing Diet booklet; and the Facebook Group will be shown how to use the Facebook group to access the weight management program, as well as how to interact with other group members, and will then be invited to join the closed Facebook group. After all of the members have joined the Facebook Group, the details of the weight management program will be posted to the group’s wall, and will consist of snapshots of all of the information from the booklet given to the Pamphlet Group, so that both the Facebook Group and the Pamphlet Group receive identical information.

**PARTICIPANT INFORMATION SESSIONS**

*Participant Information Sessions* These will be held after recruitment, and consist of three separate information sessions for the different groups, as follows:

- The Control Group will be briefly instructed to follow diet and lifestyle as explained in the Australian Dietary Guidelines and the National Physical Activity Guidelines for Adults. Also during this participant information session, the schedule of clinic appointments and the details of the questionnaires will be explained to the participants in this group.
- The Pamphlet Group will receive the weight management program in booklet form (as described in the Research Methods section) and supporting materials, such as check lists, fact sheets and shopping lists, following their baseline appointment. Also during this participant information session, the details of the weight management program will be briefly explained to the participants in this group, along with the schedule of clinic appointments and the details of the questionnaires.
- The Facebook Group will be informed that they will receive the weight management program posted to a dedicated ‘closed’ group on Facebook (as described in the Research Methods section), including supporting materials such as check lists, fact sheets, and shopping lists, following their baseline appointment. Also during this participant information session, the details of the weight management program will be explained to the participants (see Pamphlet group above), along with the schedule of clinic appointments and the details of the questionnaires. The Facebook Group will also be shown how to use the Facebook Group, as well as how to interact with other group members (such as sharing recipes, how they are integrating the program in the daily lives, forming walking groups, inspiring and motivating other, posting before and after photo of themselves, congratulating others for their successes, problem solving etc), and will then be invited to join the closed Facebook Group. In addition, the participants will be made aware of the role of the student investigator with the Facebook Group. After all of the members have joined the Facebook Group, the details of the weight management program will be added to the ‘Photos’ tab of the group as jpeg images, the equivalent of one page from the booklet per image.

At the conclusion of each participant information session, participants will be invited to ask questions, or raise any concerns that they may have.

**WEIGHT MANAGEMENT PROGRAM ON THE** **FACEBOOK GROUP**

The study investigator will be the administrator and facilitator of the Facebook Group. The group facilitator will spend two hours per week monitoring the Facebook site. Participants will be advised that the facilitator will have a presence within the site, and will monitor posts to makes sure that they fall within the guidelines specified at the Participant Information Session explained here and stated in hard copy on the Participant Information Sheet.

After all of the participants have joined the Facebook Group, snapshots of the entire weight management program will be posted on the Facebook Group wall. The weight management program will be reposted at weekly intervals, as described earlier in this proposal. This will save participants the trouble of scrolling through the more recent posts and comments, added over time, to review the program when needed. Being able to easily access the program also enables participants in this group the same advantage as the Pamphlet Group, who are able to read and review the program in hard copy at their leisure.

To stimulate discussion between Facebook Group members a list of general questions will be created. Once a week, the facilitator will post one of the questions from the list to the group’s wall. Examples of the types of questions to be posted include: Does anyone have a favourite recipe based on the program that they would like to share with the group? And, what is a good way to incorporate thirty minutes of moderate physical activity into the day? Without directly telling the participant what to do on the Facebook site, it is hoped that this minimal facilitation will help spontaneous discussions begin to take place during the course of the twelve-week intervention period as well.

Participants using Facebook will be instructed to be polite in their interactions with other members, and never to say anything that may embarrass other members, or clutter up the newsfeed with trivial information like “Sitting down for my morning coffee”. They will be instructed to post their thoughts on the weight management program and how they are integrating it into their lives, any achievements brought about by using the program, or ask for assistance from group members if they encounter a problem. Participants in this group will be instructed to offer praise and encouragement for the achievements of others, offer useful tips, form walking groups, post recipes or motivational quotes. It will be made extremely clear to the participants using Facebook that this group is in place to provide a friendly and supportive environment for all members.

The Facebook Group facilitator will also monitor the comments posted by the group daily, and will act on any negative comments or erroneous statements posted on the group’s wall with an appropriate response or correction. In the event of an offensive comment, the facilitator will remove the post immediately, and contact the offending participant privately. If the issue cannot be resolved or there is a repetition of the offence, the offending participant will be removed from the group, and will take no further part in this project.

**Privacy**: The Facebook Group will be set as a ‘Closed Group’, whereby only group members can see the group, who’s in it and what members post. In addition, the group’s administrator will be the only member to grant approval to new members. (These settings are features provided by Facebook.) These precautions will be taken to provide a discreet online environment for the participants in the Facebook Group, and also to ensure that no-one else can see or join this group.

**ASSESSMENTS**

All participants will be required to attend regular clinical appointments (for a duration of 10 minutes), as follows: at baseline, at weeks 6, 12, 18 and 24. Prior to these appointments, participants will complete the following range of questionnaires (see list below and Trial Protocol Part 3). At each clinical appointment, weight and body fat percentage (UM-018 Digital Scales; Tanita Corporation, Tokyo, Japan) will be recorded in light clothing without shoes. Height will be measured to the nearest 0•1 cm using a stadiometer (26SM 200 cm SECA, Hamburg, Germany) without shoes. Waist circumference will be measured in the standing position at the narrowest area between the lateral lower rib and the iliac crest, and hip circumference at the widest area across the buttocks. Fasting blood glucose will be taken using the Accu-Chek® Performa glucometer and lancing device (Roche Diagnostics), and blood pressure will be measure using the Automatic Blood Pressure Monitor (Omron Australia). Participants will attend their local PathWest Collection Centre to have fasting blood samples taken to measure blood lipids and blood insulin at baseline, weeks 12 and 24. Analysis will be conducted at PathWest Laboratory Medicine.

**Outcome Measures**

The primary outcome measure for this study is weight loss. Secondary outcome measures include blood glucose, blood pressure, waist circumference, as indicators of changes to cardiometabolic disease risk factors. Secondary outcome measures also include fasting blood lipids and fasting blood insulin, the latter being used with the fasting blood glucose measurements collected in the clinic to generate HOMA-IR Score. Also monitored will be hip circumference, physical activity, dietary intake as well as psychological factors (see below). These clinical measurements, and the food diary, physical activity record and questionnaires have been chosen to compare the outcomes of using the two different program delivery methods (ie written information only or Facebook).

**Clinical Measurements**

- Height (measured at Week 0 only)
- Weight
- Body fat percentage^◆^
- Waist circumference^◆^
- Hip circumference ^◆^
- Fasting blood glucose^◆^
- Blood pressure^◆^
- Blood lipids (for TC, LDL-C, HDL-C, TGs)^◆❄^
- Blood Insulin (used with FBG to calculate HOMA-IR Score)^◆❄^

^◆^These measures will be used to monitor changes that may not be reflected by a change in body weight.

^❄^These measures will be collected and analysed by PathWest Laboratory Medicine WA.

**Questionnaires**

- *Three-day Food Diary* to record participants’ food and drink intake over three consecutive days, used to measure adherence to the dietary guidelines.
- *Bouchard’s Physical Activity Record* to provide a minute, three-day snapshot of daily physical activity, and for G2 and G3 participants includes three-day step count at the end of each for the recording period, as noted in the Project Description.
- *Three-Factor Eating Questionnaire* to provide a measure of dietary restraint, disinhibition and hunger, used to assess participants’ awareness of the impulses surrounding food consumption.
- *Self-Efficacy Scale* to provide a measure of participants’ ability to achieve a desired result.
- *WHO Quality of Life Questionnaire* to provide a measure of participants’ quality of life, differentiated into four separate domains: physical health, psychological, social relationships (includes social support), and environment.
- *Depression Anxiety Stress Scale* (or *DASS 21*, the short version) provides a measure of participants’ general psychological wellbeing.
- *Self-Control Scale* provides a measure of participants’ ability to inhibit detrimental impulses.
- *Facebook Intensity and Network Density Scale* to determine the degree to which FG participants make use of Facebook, and to measure the strength and frequency of online social interactions, for FG participants only.
- *Social Media Survey* to provide a measure of participants’ opinions about social media, for FG participants only.
- *Survey of Weight Management* *Program* to provide a measure of participants’ opinions of the weight management program, specific to group allocation.
- *Diet and Physical Activity Survey* to provide a measure of participants’ intentions with regard to the dietary and physical activity modifications indicated within the weight management program. A separate version for FG participants includes a section regarding the use of Facebook to access the weight management program.
- *Personality and Individual Differences Questionnaire* measures personality traits that may influence participants’ capacity to integrate lifestyle changes, such as ingenuity, cognitive ability, insight, health anxiety, initiative, competence and happiness.
- Facebook Group page will be printed periodically to corroborate self-reported Facebook usage data.

**STATISTICAL ANALYSIS**

The results will be analysed using a mixed repeated measures ANOVA design. Data will be expressed as mean (±SEM), and statistical significance will be considered at p<0.05. All statistical analysis will be performed using SPSS 22.0 for Windows (SPSS Inc., Chicago, IL). Power Analysis: Based on a three group study with repeated measures, the ability to detect a medium to large effect size (Cohen’s d = 0.4), that is a difference of 7% initial body weight between the two intervention groups, and an alpha of 0.05, a sample size of 96 achieves 80% statistical power. To allow for an attrition rate of 20%, a total of 120 participants will be recruited.

**ETHICAL ISSUES**

Personal information will be collected from the participants during the initial screening for recruitment, as well as in the form of questionnaires administered throughout the duration of the study. Participants will be required to provide written informed consent and will be able to withdraw from the project at any time. Participants will not be identified by name in any published material, and they will be randomly assigned a code number for statistical analysis purposes.

**DATA STORAGE**

Participants' personal information will be stored in a secure, locked filing cabinet and password protected computer for 25 years after the thesis has been published and approved, as recommended by the Curtin University Human Research Ethics Committee.
