# Supplementary material for: Effects of a weight management program delivered by social media on weight and metabolic syndrome risk factors in overweight and obese adults: A randomised controlled trial
Source: PLoS One. 2017 Jun 2;12(6):e0178326. doi: 10.1371/journal.pone.0178326 (PMC5456050; doi:10.1371/journal.pone.0178326)
Supplement: S4 File — Questionnaires. (DOCX) [file pone.0178326.s004.docx]

**How to keep a Food Record**

**GENERAL HINTS**

- Include **3 days of your food intake**, consisting of 2 weekdays and 1 weekend day;
- Record **all foods and drinks** that are being consumed over the 3 days;
- Each day begins at **12 midnight;**
- Record the food **immediately** after consuming it, don’t wait until the end of the day as it’s easy to forget snacks, drinks etc;
- Remember to include **brand names** of products, **cooking methods**, and **quantities** consumed as accurately as possible;
- If possible, write down the kJ content of any frozen or pre-prepared meals, eg, Lean Cuisine, meal replacements etc;
- Please provide **food labels or nutritional value information** of foods consumed if possible;

#### REPORTING THE AMOUNTS OF FOOD

# Please weigh everything if possible;

# Ensure that the raw weight of food is reported where possible. If not, indicate that cooked weight was reported;

# If food cannot be weighed, use metric measures (teaspoon, tablespoon or cup) or dimensions to describe amount of food. *For example: 1 tablespoon of honey, Grilled T-bone steak (5cm thick x7cmx10cm).* Please find portion size estimation at the end of this document;

# When a meal is made up of several food items, each item needs to be weighed and recorded in detail separately.

# When cooking from recipes, please provide us the recipe (quantities and ingredients). Also state how many serves the recipe provides and how many serves of that recipe you consumed. See example of cheese sauce in Section G);

#### WHAT TO DO WHEN EATING OUT

- It is **OK** to eat out if this is your usual lifestyle;
- Report as much detail on your meal as possible;
- Use **metric measures** or **dimensions** of food to describe portion sizes, eg, bread roll 10 cm;
- If a mixed dish is consumed of which you do not know the exact ingredient, then provide the **menu description of the dish** and indicate the portion size of the entire meal using metric measure;
- Remember to report all **beverages** consumed including water. For example: 2 glasses of cabernet – please state if small, medium or large if you are unable to measure it;
- Include dinner rolls (with spreads), desserts, tea and coffee and after-dinner mints;

**DESCRIBING YOUR FOODS**

# Breads

*Example: Helga’s light rye bread (40g); Vitalite canola margarine (10g); IXL strawberry jam (20g)* Report **brand names**

- Identify **types (***E.g.: white, multigrain, wholemeal or continental breads)*
- Report **weight** of breads prior to toasting
- Describe **spreads** added (*E.g.: margarine, peanut butter, jam, honey)*

#### Cereals

*Example: Kellogg’s All Bran (40g); Light Start Reduced Fat milk (200g); Sugar (20g)*

- Report **brand names**
- Describe **milk and sugar**, if added to breakfast cereals
- Report **weight** of cereals prior to cooking (*E.g.: oats*)

# Pasta, rice and noodles

*Example: Barilla wholemeal pasta, boiled (100g); Bertolli virgin olive oil (20g)*

- Identify **types** (*E.g.: white or brown rice, long grain, basmati, wholemeal pasta).*
- Report **weight** prior to cooking
- Report specific **cooking methods** (*E.g.: Sunrise long-grain white rice steamed)*
- Indicate amount and type of **oil/fat** added to rice, pasta and noodles

# Fruits (including juice)

*Example: Golden Circle tropical fruit salad, canned, in natural juice, not drained (100g); Red apple, cored and peeled (50g); Berri orange juice, unsweetened (100g)*

# Identify if fresh, frozen, canned, sweetened or unsweetened

# Indicate whether canned fruit is in heavy/light syrup, natural juice, drained or undrained

- Indicate if peeled or unpeeled

# Vegetables

*Example: McCain’s frozen baby peas, boiled (100g); Devondale butter (20g)*

# Identify if fresh, frozen, canned

# Report the weight prior to cooking (raw weight)

- Report specific **cooking methods** (*E.g.: steamed, boiled, fried with oil)*
- Indicate amount and type of **oil/fat** added

# Milk

*Example: Light Start reduced fat milk (200g); Master’s light chocolate milk (500g); Tea (100g); Pura Hi-Lo milk (1.4% fat) (20g); Sugar (5g)*

- Report brand names of products used.
- Identify milk as full cream, 2% fat (Hi Lo), skimmed/non-fat, flavoured (chocolate, coffee etc)

# Report milk added to hot beverages

# Yoghurt and cheese

*Example: Ski Diet strawberry yoghurt (200g); Cheese sauce (serves 4, had 1); Mainland’s chedder cheese (110g); Light Start reduced fat milk (600g); Butter (40g); Plain flour (40g)*

# Indicate whether yoghurt are *diet*, *low-fat*, *with fruit* or *without fruit*

- Report yoghurt or cheese added to cooking

# Meat

*Example: T-bone steak, bone-intact, fried (200g) Bertolli olive oil (50g); ETA vegetable oil (50g); Gravox mushroom gravy mix (20g); Water ( 50g); Chicken breast fillet, crumbed, fried (100g)*

# Indicate the cuts of meat used

# Be sure to indicate whether bone is present and whether visible fat and skin has been removed

- **Report the** weight **prior to cooking (raw weight) & make a note that it is the raw weight**

# Indicate amount and type of oil/fat added. For example: chicken crumbed and deep fried with 30ml of Crisco canola oil

# Indicate what type of cooking method was used to prepare meats. For example grilled or BBQ T-bone steak.

# Specify whether gravy or other sauces are added to meat dishes

# Nuts and Legumes

# Indicate if legumes are dried, fresh, frozen or canned

- Indicate if nuts are roasted, salted, raw, chocolate-coated or candied

### J) Extra foods

*Example: Pizza Hut Meat Lover’s pizza, thick crust (200g); McDonald’s Big Mac, extra cheese (200g)*

- These include all snack foods, fast foods, pies, pasties, pizza, fries, lollies, biscuits, cakes, pastries, ice-cream, sugar, oils
- Please provide **food labels** or **nutritional value information** of foods consumed where possible

# Include brand names of products

# Describe flavour and variety of fast food in detail

# Report nibbles and snacks as soon as you have consumed them so that you don’t forget

# *Remember to report*: sugar and milk in tea and coffee; spreads on breads and crackers; dressings, gravies and oils added to salads and other foods.

**Tools to estimate portion size**


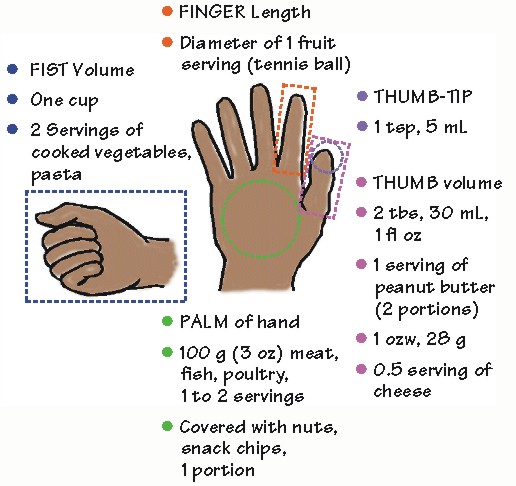

**Instructions for Recording Physical Activity**

Please record all the physical activity you do for three days in a row, consisting of two week days and one week-end day.

Use the activity coding list to accurately record the number that reflects the type of physical activity you completed.

*Time* In the box next to the correct time, record the activity code that corresponds to the level of physical activity you carried out during each 5 minute period.

*Activity* If an activity is carried out over a long period of time (eg sleeping or cooking) then you can draw a continuous line in the rectangular boxes until there is a change in activity.

Please see the completed example on the next page of these instructions.

| Activity Code | Example of Activity for each code | |
| --- | --- | --- |
| 1 | Lying down | Sleeping, resting in bed |
| 2 | Seated | Sitting, listening in class, eating, writing by hand or typing, taking a bath, reading, listening to the radio or T.V. |
| 3 | Light standing activity | Washing, shaving, combing hair, dusting, cooking. |
| 4 | Light moving activity | Slow walk (strolling), driving a car, getting dressed, taking a shower |
| 5 | Light manual work | Housework (washing windows, floor sweeping etc), tailor, baker, labour work, painting, waiting on tables, nursing chores, doing the bed, moderately quick walking (going to school, shops). |
| 6 | Light sport of leisure activities | Baseball, table tennis, golf, croquet, sailing, cycling (leisure), volleyball, canoeing or rowing, archery |
| 7 | Moderate manual work | Carpentry, house building, wood cutting, loading and unloading bags or boxes |
| 8 | Moderate sport or leisure activities | Badminton, cycling (race bike), dancing, tennis, jogging (slow running), swimming, horseback riding, brisk walking. |
| 9 | Intense manual work:  Intense sport or leisure activities: | Cutting tree branches  Running in a race, squash, basketball, football |

*Accuracy* Circle the number that best explains % accuracy of each day of your physical activity record. If necessary, please leave a comment that explains the accuracy of that day’s record.

*Example* In the example below, the *activity code* number **1** was written at **12:00 am**. A line was drawn from 12:00 am time to **6:25 am**. This shows that between 12:00 am to 6:25 am this person was sleeping and/or resting in bed.

Then at **6:30 am** the *activity code* number **3** was written with a line drawn to **6:40 am**, which shows that this person was standing from 6:30 to 6:40 am.


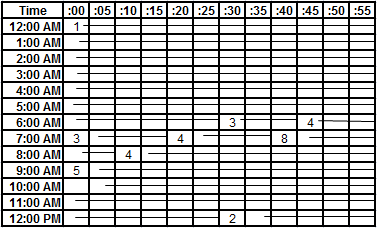


Please keep these instructions in your folder and refer to them each time you complete your physical activity record. ☺

**Physical Activity Record**

Participant ID:____________

Date:___________________


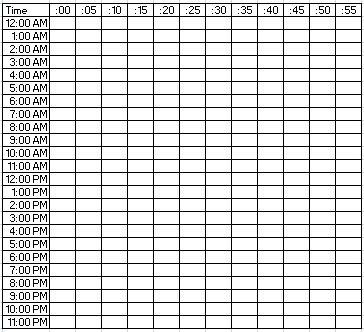


**Please circle:**

A. Today is Consecutive Day number:

1 2 3

B. Day of the week:

Mon Tues Weds Thurs Fri Sat Sun

**STEP COUNT:**

Day 1: _____________

Day 2: _____________

Day 3: _____________

| Activity Code | Example of Activity for each code | |
| --- | --- | --- |
| 1 | Lying down | Sleeping, resting in bed |
| 2 | Seated | Sitting, listening in class, eating, writing by hand or typing, taking a bath, reading, listening to the radio or T.V. |
| 3 | Light standing activity | Washing, shaving, combing hair, dusting, cooking. |
| 4 | Light moving activity | Slow walk (strolling), driving a car, getting dressed, taking a shower |
| 5 | Light manual work | Housework (washing windows, floor sweeping etc), tailor, baker, labour work, painting, waiting on tables, nursing chores, doing the bed, moderately quickly walking (going to school, shops). |
| 6 | Light sport of leisure activities | Baseball, table tennis, golf, croquet, sailing, cycling (leisure), volleyball, canoeing or rowing, archery |
| 7 | Moderate manual work | Carpentry, house building, wood cutting, loading and unloading bags or boxes |
| 8 | Moderate sport or leisure activities | Badminton, cycling (race bike), dancing, tennis, jogging (slow running), swimming, horseback riding, brisk walking. |
| 9 | Intense manual work:  Intense sport or leisure activities: | Cutting tree branches  Running in a race, squash, basketball, football |

**Accuracy of Physical Activity Log:**

%: 0 10 20 30 40 50 60 80 90 100 accurate

**If today’s physical activity log was inaccurate, please describe:**

______________________________________________________________________________________________________________________________________________________________________________________________

_______________________________________________________________________________________________


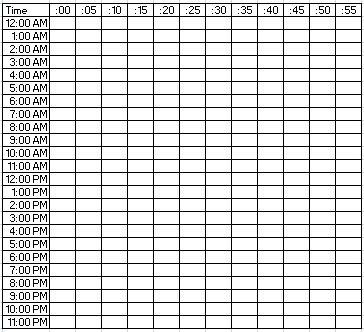

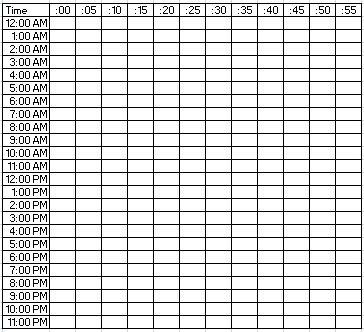


**Please circle:**

A. Today is Consecutive Day number:

1 2 3

B. Day of the week:

Mon Tues Weds Thurs Fri Sat Sun

**Please circle:**

A. Today is Consecutive Day number:

1 2 3

B. Day of the week:

Mon Tues Weds Thurs Fri Sat Sun


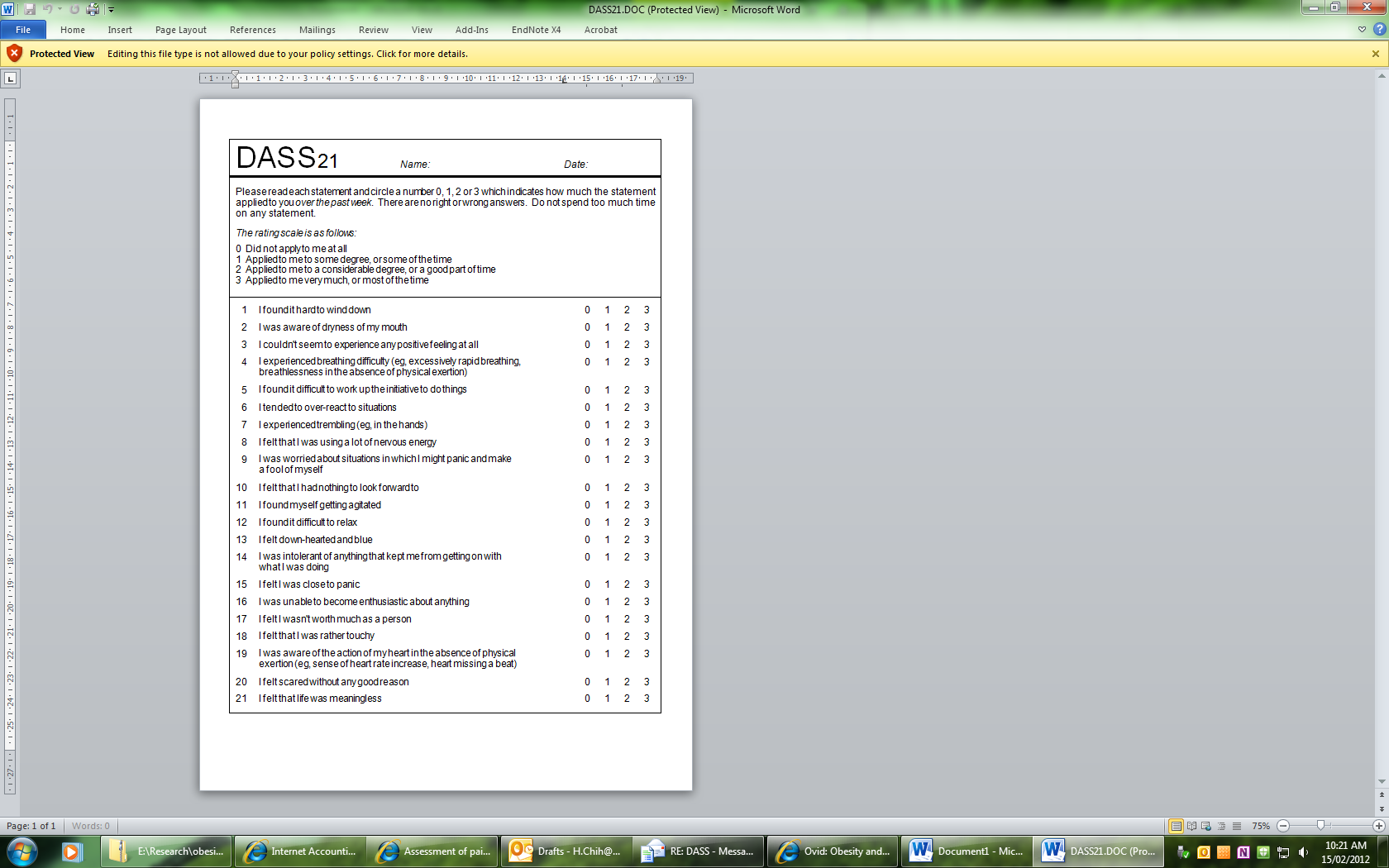


*Participant ID Number*:

**Your Opinions About Diet and Physical Activity**

**Date:**___________________ **Participant ID Number:**___________

Please fill in a number for each question that corresponds to your agreement with the following statements:

**1** = Strongly agree

**2** = Agree

**3** = Neither agree nor disagree

**4** = Disagree

**5** = Strongly disagree

Please note: Everybody feels differently, so there are no right or wrong answers to these questions. I am interested to know your opinions about healthy eating and doing regular physical activity, as recommended in the dietary and physical activity guidelines provided.

**Part One: These questions refer to your opinions about following the dietary guidelines provided**

______A. It will be good for me to eat a healthy diet over the next 6 to 12 weeks

______B. It will be satisfying for me to follow a healthy diet over the next 6 to 12 weeks

______C. It will be easy for me to follow the dietary guidelines over the next 6 to 12 weeks

______D. There is a lot I can do to make sure I follow the dietary guidelines over the next 6 to 12 weeks

______E. I plan to follow the dietary guidelines provided over the next 6 to 12 weeks

______F. I intend to follow the dietary guidelines provided over the next 6 to 12 weeks

______G. Most people who are important to me would want me to eat a healthy diet over the next 6 to 12 weeks

______H. People I know would approve of me eating a healthy diet over the next 6 to 12 weeks

**Part Two: These questions refer to your opinions about following the physical activity guidelines provided**

______A. It will be good for me to do regular physical activity over the next 6 to 12 weeks

______B. It will be satisfying for me to do regular physical activity over the next 6 to 12 weeks

______C. It will be easy for me to follow the physical activity guidelines provided over the next 6 to 12 weeks

______D. There is a lot I can do to make sure I follow the physical activity provided over the next 6 to 12 weeks

______E. I plan to follow the physical activity guidelines provided over the next 6 to 12 weeks

______F. I intend to follow the physical activity guidelines provided over the next 6 to 12 weeks

______G. Most people who are important to me would want me to do regular physical activity over the next 6 to 12 weeks

______H. People I know would approve of me doing regular physical activity over the next 6 to 12 weeks

Thank you for your time and effort ☺

**Facebook Intensity and Network Density Scale**

**Date:**_____________________ **Participant ID Number:**_________

Please fill in a number for each question that corresponds to your agreement with the following statements:

**1** = Strongly agree

**2** = Agree

**3** = Neither agree nor disagree

**4** = Disagree

**5** = Strongly disagree

**Part One: These questions refer to the Facebook Group in this study**

_______1. The Facebook Group is part of my everyday activity

_______2. I like to tell people I’m part of a Facebook Group

_______3. I feel out of touch when I haven’t logged onto the Facebook Group for a while

_______4. I feel I am part of the Facebook Group community

_______5. The Facebook Group helps me to continue with the weight management program

_______6. I would be sorry if the Facebook Group shut down

**Part Two: These questions refer to the members of the Facebook Group in this study**

_______1. There are a lot of interactions among Group members

_______2. Group members share frequent communications

_______3. Group members frequently discuss common problems

_______4. Group members encourage each other

_______5. At least one Group member has helped me with the weight management program

_______6. I feel comfortable sharing my opinions about the weight management program with Group members

Please note: Your answers to all of these questions will not be shared with the other Group members.

Thank you for your time and effort ☺

**Self-Control Scale**

**Date: ____________________ Participant ID: _______________**

This survey asks you a little about yourself. Please indicate (by circling a number) how much each of the following statements reflects **how you typically are**. Everyone feels differently about this so there are no right or wrong answers, we are interested in your ***opinions***. Do not spend too long on any one statement. All responses are strictly ***confidential***, and please answer ***all the questions*.**

1 = not at all like me

2 = unlike me

3 = sometimes like me

4 = like me

5 = very much like me

1. I have a hard time breaking bad habits. 1 2 3 4 5
2. I am lazy. 1 2 3 4 5
3. I say inappropriate things. 1 2 3 4 5
4. I do certain things that are bad for me, if they are fun. 1 2 3 4 5
5. I refuse things that are bad for me. 1 2 3 4 5
6. I wish I had more self-discipline. 1 2 3 4 5
7. I am good at resisting temptation. 1 2 3 4 5
8. People would say that I have iron self-discipline. 1 2 3 4 5
9. Pleasure and fun sometimes keep me from getting work done. 1 2 3 4 5
10. I have trouble concentrating. 1 2 3 4 5
11. I am able to work effectively toward long-term goals. 1 2 3 4 5
12. Sometimes I can't stop myself from doing something,

even if I know it is wrong. 1 2 3 4 5

1. I often act without thinking through all the alternatives. 1 2 3 4 5

**Self-Efficacy Scale**

Date:____________________ Participant ID Number:______________

Please circle the number that most appropriately corresponds to the following statements:

|  | Not true at all | Barely true | Moderately true | Exactly true |
| --- | --- | --- | --- | --- |
| 1. I can always manage to solve problems if I try hard enough | 1 | 2 | 3 | 4 |
| 2. If someone opposes me, I can find means and ways to get what I want | 1 | 2 | 3 | 4 |
| 3. It’s easy for me to stick to my aims and accomplish my goals | 1 | 2 | 3 | 4 |
| 4. I am confident that I could deal efficiently with unexpected events | 1 | 2 | 3 | 4 |
| 5. Thanks to my resourcefulness, I know how to handle unforeseen situations | 1 | 2 | 3 | 4 |
| 6. I can solve most problems if I invest the necessary effort | 1 | 2 | 3 | 4 |
| 7. I can remain calm when facing difficulties because I can rely on my coping abilities | 1 | 2 | 3 | 4 |
| 8. When I am confronted with a problem, I can usually find several solutions | 1 | 2 | 3 | 4 |
| 9. If I am in a bind, I can usually think of something to do | 1 | 2 | 3 | 4 |
| 10. No matter what comes my way, I’m usually able to handle it | 1 | 2 | 3 | 4 |

Thank you ☺

**Your Opinions About Social Media**

**Date:**______________________ **Participant ID:** ______________

I am interested in your opinions about social media. Remember, when I talk about “social media” I mean social networking and micro-blogging sites like Facebook, MySpace, Twitter, Instagram etc.

**PART ONE**

Please circle the correct answer for the following question/s

1. I have an account on one or more social media site (eg. Facebook, MySpace, Twitter, Instagram etc).

Yes / No

2. If you answered ‘yes’ to Question 1, please circle how often you accessed your social media account/s in the last four weeks.

More than once daily / Once daily / Several times a week / Once a week / Less than once a week / Not at all

**PART TWO**

Please fill in a number that corresponds to your agreement with the following statements about social media. There are no right or wrong answers to these questions.

_______A. Social media is a good invention

**1** = Strongly agree

**2** = Agree

**3** = Neither agree nor disagree

**4** = Disagree

**5** = Strongly disagree

_______B. Social media is time consuming

_______C. Social media is a good way to share information

_______D. Social media helps people stay in touch with each other

_______E. Privacy is properly protected on social media

_______F. Social media is difficult to use

_______G. I enjoy using social media

_______H. I use social media mainly because people I know use it

_______I. I like being part of an online community

_______J. Social media fills a gap in my offline social network

_______K. I like sharing my interests or concerns on social media

_______L. I often find others on social media with similar concerns or interests

_______N. Social media provides me with a sense of moral support

Thank you for completing this survey ☺

**Survey of Weight Management Program**

**Date:** _______________ **Participant ID:** _________

Please tell us your opinions of the weight management program used in this study.

**1** = Strongly agree

**2** = Agree

**3** = Neither agree nor disagree

**4** = Disagree

**5** = Strongly disagree

Please rate the following statements according to the numbered scale (above):

_____A. Overall, I am happy I participate in this study

_____B. I found the physical activity guidelines easy to follow

_____C. I found the dietary guidelines easy to follow

_____D. Having the daily allowance of food divided into food groups made it easy to incorporate the eating plan into my daily life

_____E. Having the food groups divided into ‘units per day’ or ‘serves per day’ made it easy to incorporate the daily allowance into my daily meals

F. How often did you need to refer to the dietary guidelines? Please circle

1. For every meal, when grocery shopping and before eating out
2. For every meal and when grocery shopping
3. For every meal and before eating out
4. For every meal only
5. Twice a day
6. Once a day
7. Once a week

G. How often did you need to refer to the physical activity guidelines? Please circle

1. More than twice a day
2. Twice a day
3. Once a day
4. Once a week
5. Once only

Thank you ☺
