# Supplementary figures and images for: Effects of a weight management program delivered by social media on weight and metabolic syndrome risk factors in overweight and obese adults: A randomised controlled trial
Source: PLoS One. 2017 Jun 2;12(6):e0178326. doi: 10.1371/journal.pone.0178326 (PMC5456050; doi:10.1371/journal.pone.0178326)

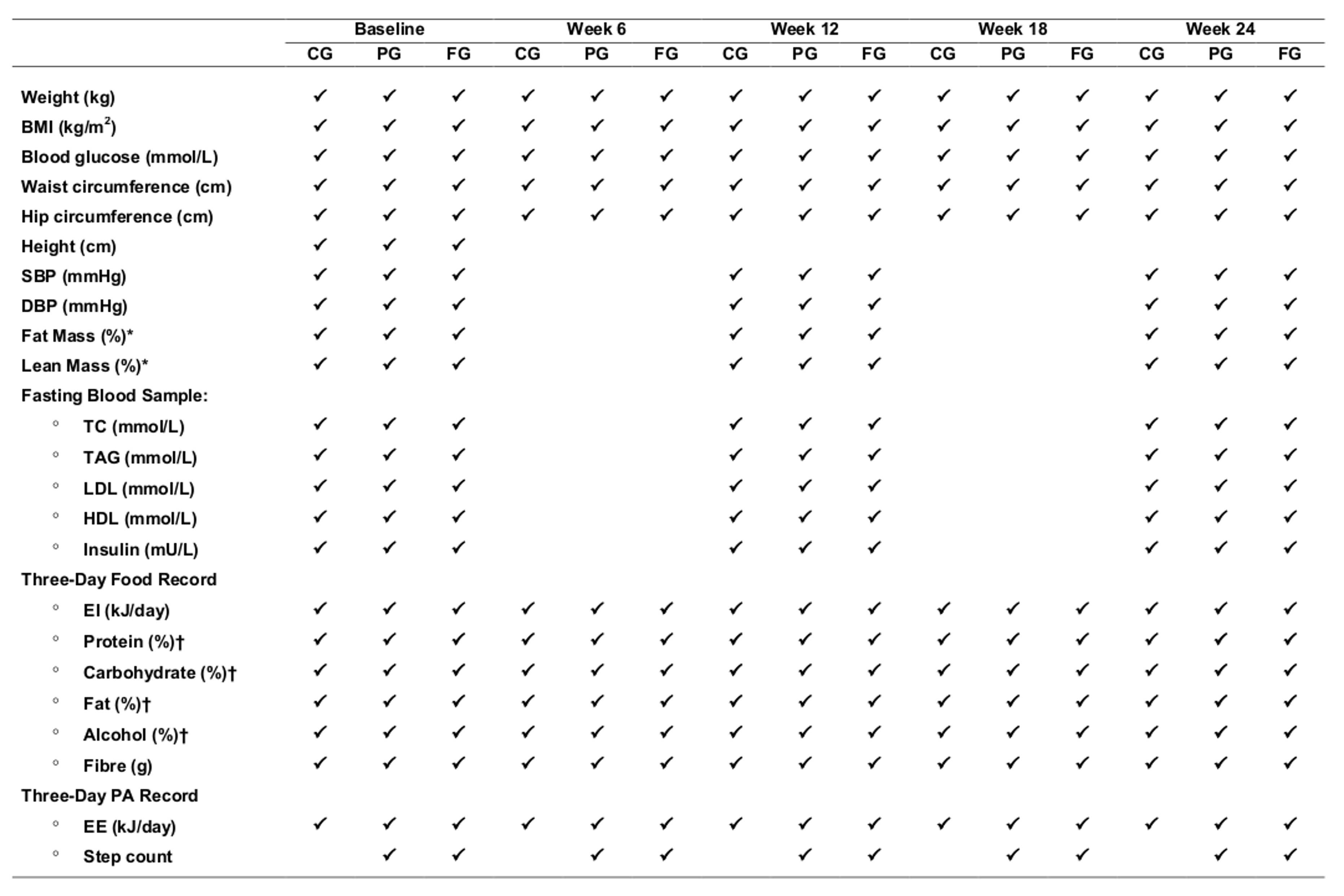

Supplement: S1 Fig — (TIF) [file pone.0178326.s005.tif]
